# Evolved DNA Duplex Readers for Strand-Asymmetrically Modified 5-Hydroxymethylcytosine/5-Methylcytosine CpG Dyads

**DOI:** 10.1021/jacs.1c10678

**Published:** 2022-02-14

**Authors:** Benjamin C. Buchmuller, Jessica Dröden, Himanshu Singh, Shubhendu Palei, Malte Drescher, Rasmus Linser, Daniel Summerer

**Affiliations:** † Faculty of Chemistry and Chemical Biology, TU Dortmund University, Otto-Hahn-Straße 4a, 44227 Dortmund, Germany; ‡ Department of Chemistry and Konstanz Research School of Chemical Biology, University of Konstanz, Universitätsstraße 10, 78457 Konstanz, Germany

## Abstract

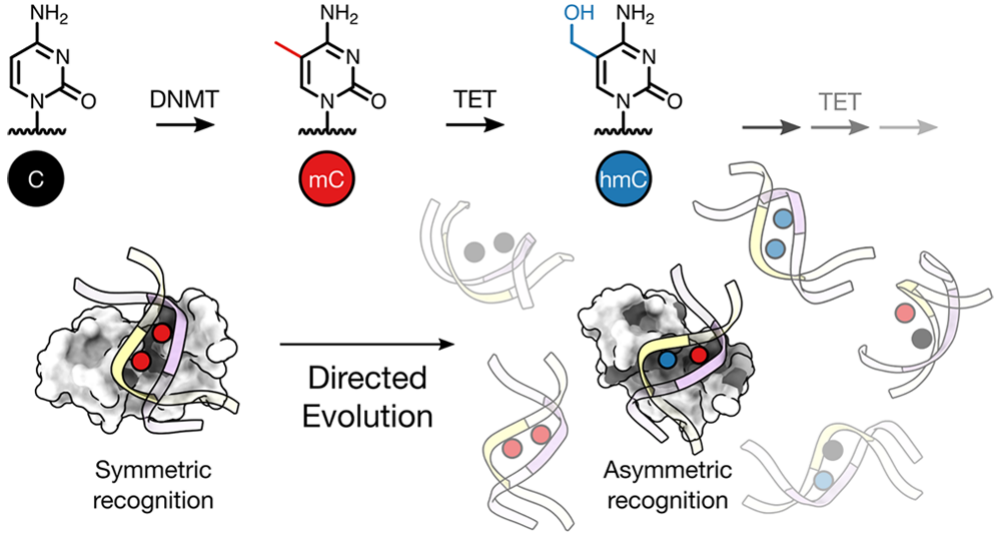

5-Methylcytosine (mC) and 5-hydroxymethylcytosine (hmC), the two main epigenetic modifications of mammalian DNA, exist in symmetric and asymmetric combinations in the two strands of CpG dyads. However, revealing such combinations in single DNA duplexes is a significant challenge. Here, we evolve methyl-CpG-binding domains (MBDs) derived from MeCP2 by bacterial cell surface display, resulting in the first affinity probes for hmC/mC CpGs. One mutant has low nanomolar affinity for a single hmC/mC CpG, discriminates against all 14 other modified CpG dyads, and rivals the selectivity of wild-type MeCP2. Structural studies indicate that this protein has a conserved scaffold and recognizes hmC and mC with two dedicated sets of residues. The mutant allows us to selectively address and enrich hmC/mC-containing DNA fragments from genomic DNA backgrounds. We anticipate that this novel probe will be a versatile tool to unravel the function of hmC/mC marks in diverse aspects of chromatin biology.

## Introduction

**Figure 1:**
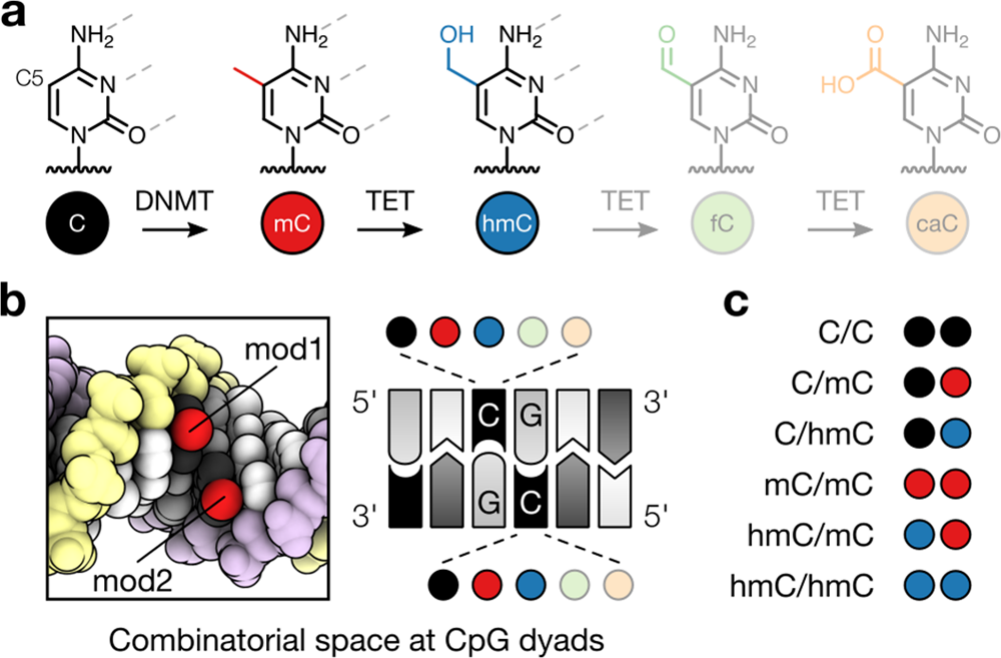
5-Modification of cytosine at CpG dinucleotides. (a) DNMT and TET enzymes convert cytosine into mC, hmC, fC, and caC. (b) Each combination of 5-modifications in the two CpG strands constitutes a unique physicochemical mark in the DNA major groove (left: mC/mC CpG in duplex DNA; Protein Data Bank (PDB) ID 329d). (c) Combinations of the most prevalent cytosine 5-modifications C, mC, and hmC.

Nature uses postsynthetic modifications of nucleic acids to control and modulate their activity by concealing or creating specific protein interaction sites. In mammalian DNA, carbon 5 of cytosine is the main site for such derivatization and gives rise to at least five physicochemically unique nucleobases (Figure 1a).^1^ Cytosine 5 substituents are presented in the DNA major groove^2^ and can decisively influence regulatory protein–DNA interactions.^3−5^ Cytosine is transformed to 5-methylcytosine (mC) by DNA methyltransferases (DNMTs) almost exclusively in CpG dyads. This methylation is maintained in a strand-symmetric state (i.e., in both DNA strands), and the resulting “mC/mC” sites (Figure 1b) play essential roles for transcription regulation, differentiation, and development.^1^ In contrast, the iterative oxidation of mC to 5-hydroxymethyl- (hmC), 5-formyl- (fC), and 5-carboxycytosine (caC) by ten-eleven-translocation dioxygenases (TETs) occurs nonprocessively. Along with active DNA demethylation and/or replication, this gives rise to diverse combinations of strand-symmetrically and strand-asymmetrically modified CpGs and creates a complex landscape of physicochemical marks in the double-stranded genome.^6−9^

Insights into the biological roles of hmC/mC and other combinations (Figure 1c) critically depend on tools for revealing their sequence position at the level of the single DNA duplex. Although modified cytosines can be selectively converted for subsequent DNA (bisulfite) sequencing analyses,^10,11^ it is not feasible to convert a specimen multiple times to specifically detect another modified cytosine nucleobase in the same DNA duplex (which also limits the potential of elegant hairpin-sequencing of single duplexes).^12,13^ Therefore, only probabilistic assessments based on averages from large molecule populations can be made about the presence of two (or more) cytosine modifications in CpG dyads of single duplexes.

Alternative approaches rely on protein-based affinity probes such as antibodies, methyl-CpG-binding domains (MBDs), and others that selectively recognize DNA fragments containing modified cytosines in diverse assays, ranging from enrichment over footprinting to cell imaging.^14−19^ However, selective probes for hmC/mC CpGs and other TET-related DNA modification combinations are missing:^3,20^ Although MBDs stand out as the only probes that recognize cytosine modifications in both strands of CpGs (i.e., mC/mC CpGs), they are repelled by oxidized mC derivatives.^21,22^

## Results and Discussion

**Figure 2:**
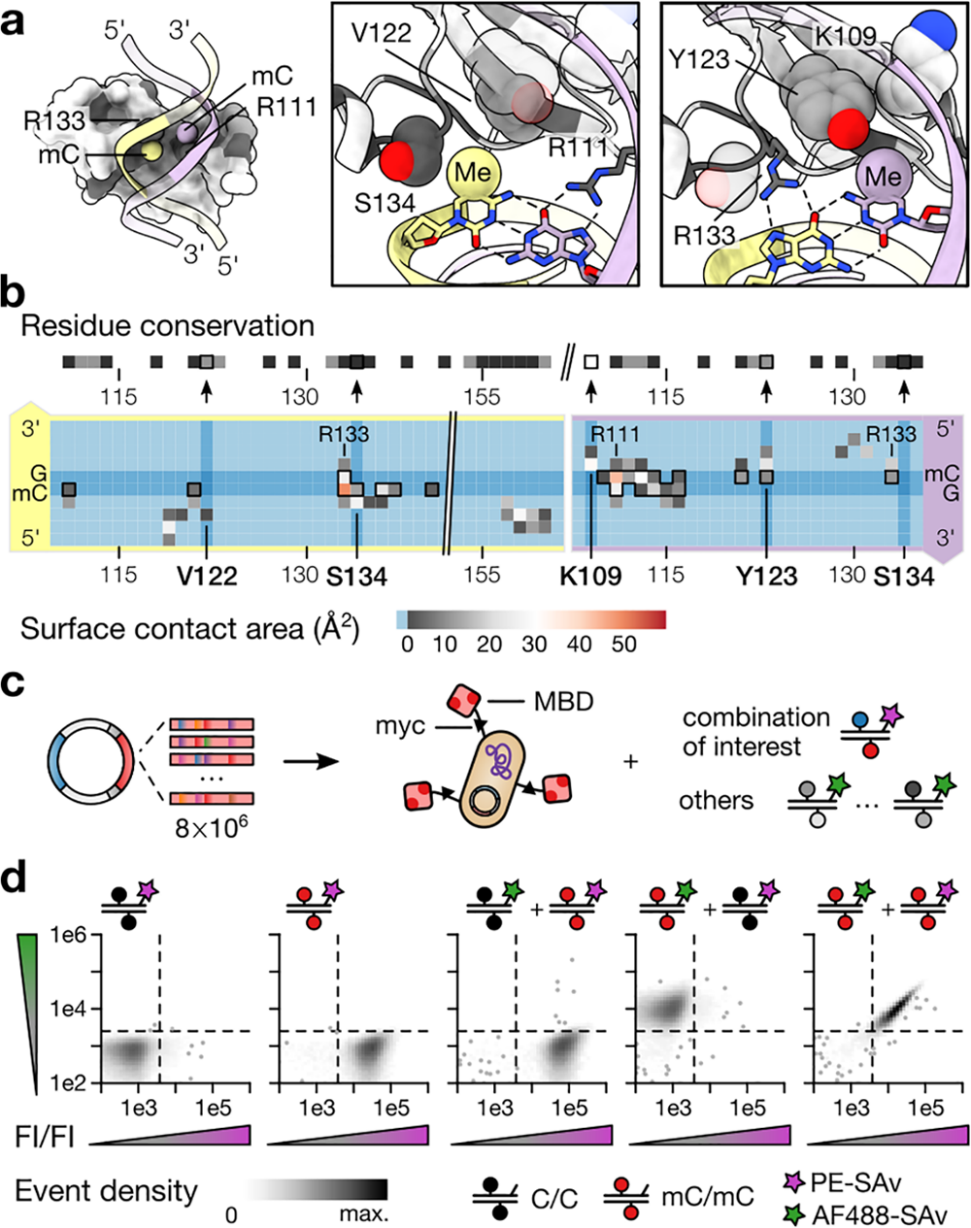
Directed evolution method for creating MBD proteins with selectivity for noncognate CpG modifications based on bacterial cell surface display. (a) Structure of the MBD of MeCP2 in complex with mC/mC DNA (PDB 3c2i);^35^ randomized residues of library as van der Waals spheres: 5-methyl groups (Me) in purple and yellow; conserved residues in dark gray. (b) Sequence conservation in top row and MBD–DNA surface contact area in the DNA binding site by strand. (c) AIDA-based bacterial surface display of an MBD library and DNA probes for FACS-selection of hmC/mC binders. (d) Validation of FACS assay with surface-displayed wt MBD2 and DNA probes that contain a single C/C or mC/mC CpG labeled with phycoerythrin (PE, magenta) or AF488 (green).

To engineer protein probes capable of recognizing strand-asymmetrically modified CpG dyads, we started from the MBD of MeCP2. This domain interacts with the two methyl groups of mC/mC CpGs via two distinct sets of amino acids (Figure 2a,b).^21−23^ We selected four residues in immediate proximity to the mC nucleobases for mutagenesis by NNK codon degeneration: (i) S134 which interacts with a 5′-phosphate in vicinity of one mC. (ii) Y123 which interacts with the 4-amino group of the other mC through a water molecule. (iii) V122/K109 which do not directly interact with mC in wild-type (wt) MeCP2 but have the potential for novel interactions when mutated.^23^ We preserved R133 and R111, which confer CpG-specificity via two methyl-arginine-guanine triads (Figure 2a,b).^23^

To screen this library of a theoretical diversity of ∼10^6^ genotypes, we developed a facile selection system that allows to rapidly assign selectivities for multiple on- and off-target CpG modifications during competitive binding to a single MBD mutant. We explored the potential of the adhesin involved in diffuse adherence-I (AIDA-I) autotransporter protein for displaying MBDs on the cell surface of *E. coli* (Figure 2c).^24,25^ This would allow for pooled, iterative sampling of MBD clones that bound to synthetic DNA probes using flow cytometry (FCM, see Figure 2c and the Supporting Information). The probes contained a single CpG with different CpG modifications in an oligo-dA/dT context and were individually labeled with different fluorophores. After establishing conditions for functional MBD display, we conducted the first model selection with wt MBDs and dsDNAs containing mC/mC or C/C CpG dyads labeled in two colors. This assay afforded a low false positive rate (<0.2–1.2%; Figures 2d and S1) and reliably separated a mixture of MBD2 and MBD3, having high and low affinity for mC/mC, respectively^21^ (>95% success rate; Figure S2). Furthermore, a selectivity profile obtained from a single-color variation of this assay matched the one obtained for wt MBD2 in electromobility shift assays (EMSA, Figure S3). This indicates that MBD selectivities can be correctly identified and characterized with our assay.

**Figure 3:**
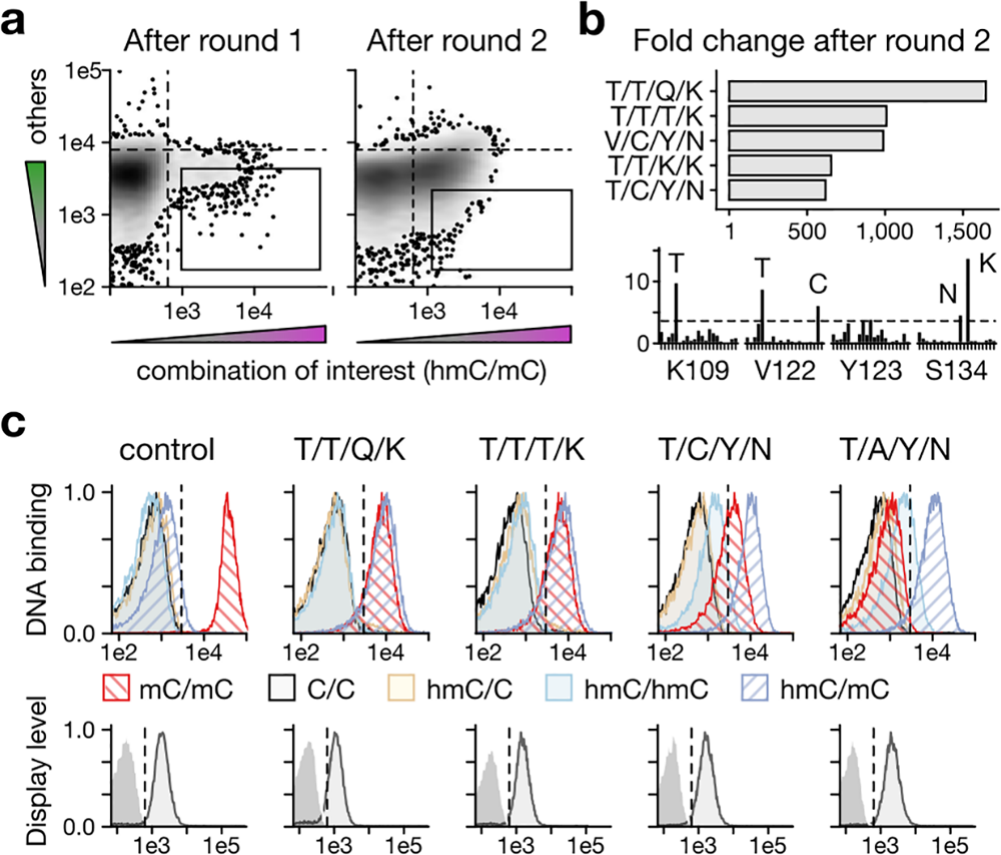
Inverting the selectivity of wt MeCP2 from mC/mC to hmC/mC CpG dyads by directed evolution. (a) Event density after successive enrichment of the MeCP2-MBD library for hmC/mC binding. (b) Phenotype enrichment (top) and diversity per degenerated residue (bottom) as determined by NGS. (c) Single-color (PE) assay for the indicated CpG modifications with selected clones and wt MBD2; display levels were assessed via anti-myc antibody (Ab) staining.

We screened our MBD library for binding to probes containing an hmC/mC CpG, the initial and presumably most abundant TET oxidation product at mC/mC CpGs.^26^ After two rounds of iterative screening for a selective hmC/mC binder in the presence of all 14 off-target CpG dyads, we observed enrichment (Figure 3a) and determined the genotypes of ∼250 clones by next-generation sequencing (NGS; Figure S4). Intriguingly, many of the clones that were enriched the highest after the second selection round shared a (i) K109T, (ii) V122T or V122C, or (iii) S134N or S134K substitution (Figure 3b, top). We observed a similar enrichment on the level of individual NNK codons analyzed for all sequenced clones. However, the randomized Y123 did not converge to a particular amino acid substitution (Figures 3b, bottom, and S4d). After a final sorting step, we individually analyzed 15 clones covering four genotypes in our single-color FACS binding assay (Figure 3c). Two phenotypes that had obliterated Y123 (T/T/Q/K and T/T/T/K) showed similar binding of mC/mC and hmC/mC, whereas two others that retained Y123 (T/C/Y/N and T/A/Y/N) bound hmC/mC more strongly than mC/mC, C/C, hmC/C, or hmC/hmC (Figure 3c).

**Figure 4:**
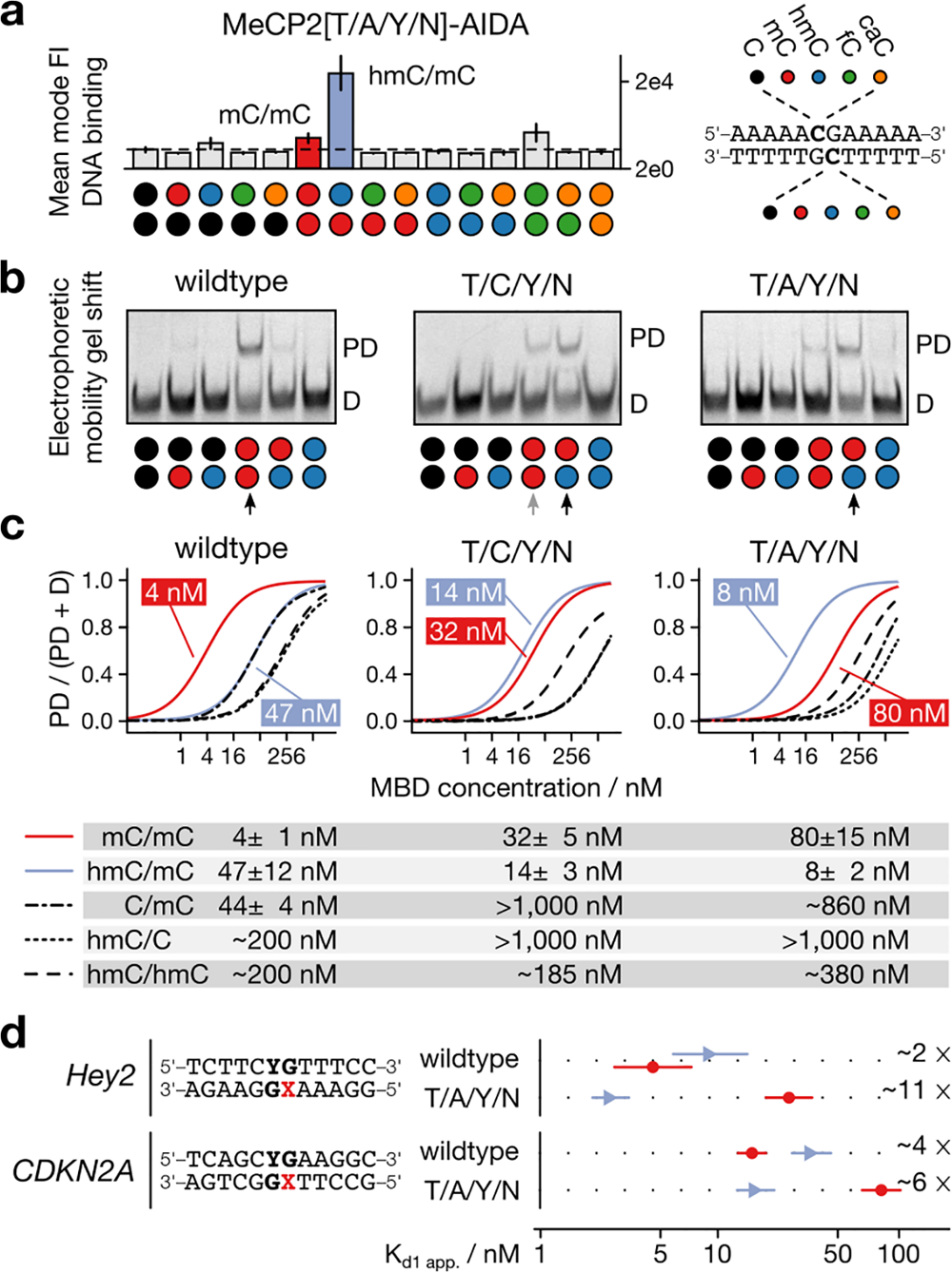
Biochemical characterization of the hmC/mC-selective reader MeCP2 T/A/Y/N. (a) Selectivity profile of MeCP2 T/A/Y/N mutant for all 15 CpG combinations assessed by display assay. (b) EMSA assay with DNA duplexes containing one CpG with a combination of C (black), mC (red), or hmC (blue) at 10 nM wt or T/A/Y/N MeCP2-MBD. D: DNA; PD: Protein–DNA complex. (c) EMSA-based apparent *K*_d_ measurements for DNA duplexes containing a single mC/mC or hmC/mC or other modified CpGs in oligo-dA/dT context or (d) natural gene sequences (see the Supporting Information).

We next measured the full selectivity profiles for MeCP2 T/A/Y/N. Remarkably, this mutant discriminated not only against the progenitor CpG dyad mC/mC but also against all 14 off-target CpG dyads, in full agreement with our challenging screening goal (Figures 4a and S5). EMSAs with recombinantly expressed and purified proteins confirmed the inverted selectivity of T/A/Y/N (and T/C/Y/N) compared to the wildtype with respect to the CpG dyads hmC/mC and mC/mC (Figure 4b). Moreover, quantitative analyses revealed for T/A/Y/N, an apparent *K*_d_ of 8 ± 2 nM for a single hmC/mC CpG, which was 9.4-fold lower than that for an mC/mC CpG in the same context (80 ± 15 nM). In comparison, T/C/Y/N showed a lower affinity and selectivity (Figures 4c and S5). However, wt MeCP2 bound the mC/mC CpG 11.8-fold more strongly than to the hmC/mC CpG (Figure 4c), in agreement with previous studies.^21,22^ Hence, a single selection round led to a 110-fold inversion of selectivity between natural progenitor and evolved mutant. Indeed, the presence of both modifications (hmC and mC) was required for the T/A/Y/N and the T/C/Y/N mutant for high-affinity binding, as the hemimodified C/mC or hmC/C were bound with significant lower affinity, thus suggesting a positive mode of recognition (Figure 4c).

As important requirement for genomic applications, we next evaluated if this selectivity was limited to the simple oligo-dA/dT context around the CpG we used in the selection or if it would be transferable to other sequence context. We conducted EMSAs with probes containing a single CpG in two different, natural vertebrate promoter sequences (Hey2 and CDKN2A).

To our delight, T/A/Y/N also showed 5.5- and 1.5-fold higher selectivity in these sequences for an hmC/mC dyad over an mC/mC dyad than the wt MeCP2 showed for mC/mC over hmC/mC (Figures 4d and S6). This data indicate that the engineered selectivity of our novel probe is not limited to a particular sequence context.

**Figure 5:**
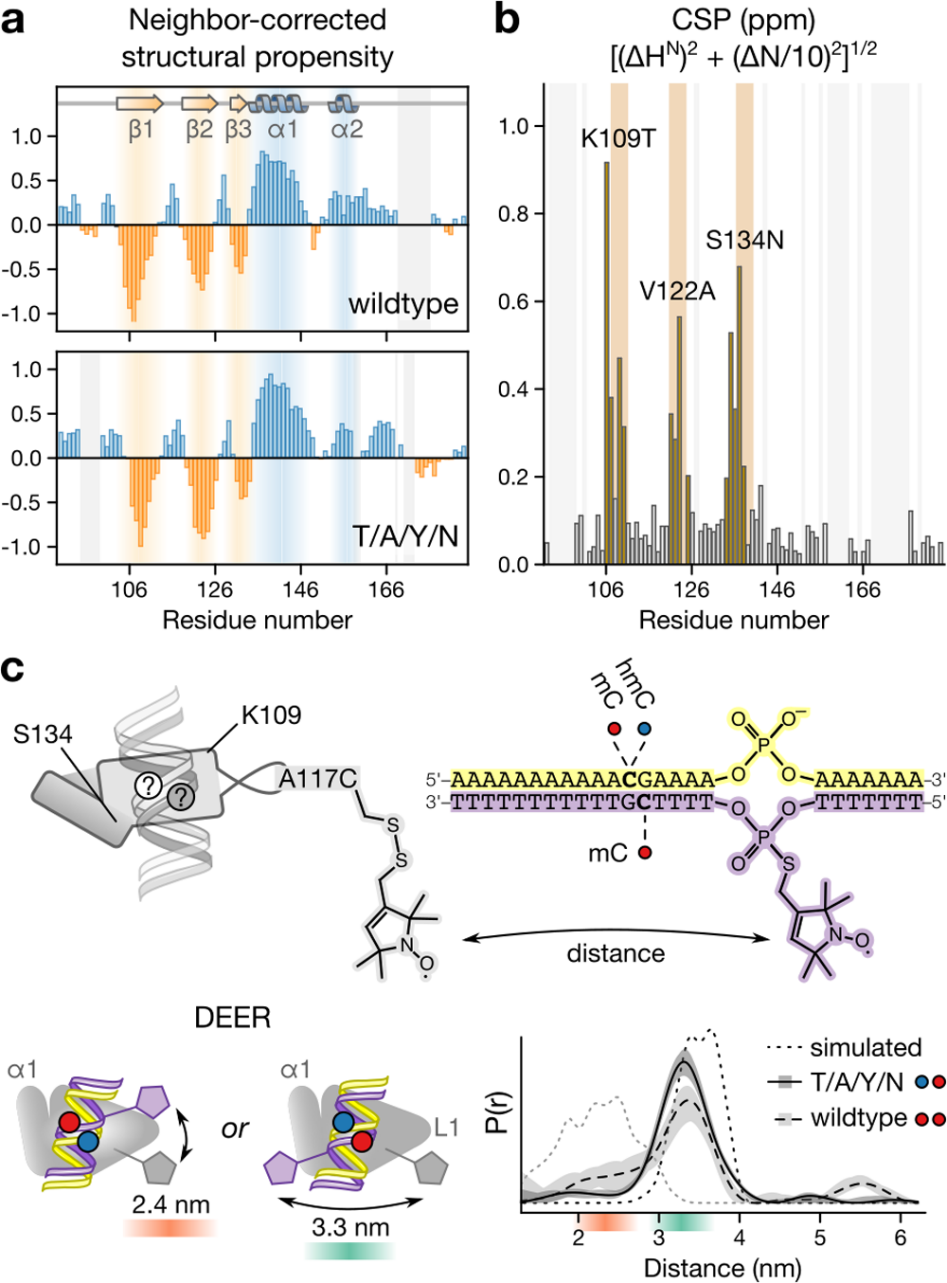
Structural characterization of the hmC/mC-selective reader MeCP2 T/A/Y/N. (a) Structural propensity of wt and T/A/Y/N MeCP2 assessed by NMR (^1^H, ^15^NH, ^13^C_α_, ^13^C_β_, and ^13^CO) chemical shifts.^31^ (b) Chemical shift perturbations (CSP) between wt and T/A/Y/N MeCP2. Location sites are marked on top. Gray shades denote overlapped H/N peaks. (c) DEER measurements and simulations^36^ with singly methanethiosulfonate (MTSL) spin-labeled A117C mutants of wt or T/A/Y/N MeCP2 in complex with singly spin-labeled DNA duplex for assessing the CpG binding orientation. Top: Labeling strategy. Bottom left: Possible orientations of DNA duplex in the MeCP2-DNA complex. Bottom right: DEER distance distributions and simulations for wt and T/A/Y/N MeCP2.

Since mutations in the MBD core structure have the potential to alter the physiological fold of the domain^27,28^ with implications for the recognition of noncognate CpG combination,^22^ we evaluated the integrity of the wildtype and the mutated scaffold in ^15^N/^13^C solution NMR. In each case, we obtained residue-specific ^1^H, ^13^C, and ^15^N resonance assignments using a suite of 3D ^1^HN-observed backbone experiments similar to previous studies.^30^ Secondary-structure propensities assessed from shifts of ^15^N, ^13^C_α_, ^13^C_β_, ^1^HH, and ^13^CO in the framework of neighbor-corrected structural-propensity prediction^31^ revealed β-strand and α-helical segments as well as short stretches of unstructured elements in line with previous MBD structures and did not differ between wt and T/A/Y/N MeCP2 (Figure 5a).^30^ Comparison of ^1^HN and ^15^N chemical shifts monitored under identical experimental conditions for wt and T/A/Y/N MeCP2 revealed that the chemical-shift differences occurred exclusively as local perturbations specific to the three mutated sites (Figure 5b). This suggests that the novel selectivity of T/A/Y/N resides in rather defined interactions involving the three residues K109T, V122A, and S134N without topological impairment.

To quickly establish which of the substituted residues resided in vicinity of the hmC and mC nucleobase, respectively, we determined the binding orientation of a DNA duplex in the MBD via EPR double electron–electron resonance (DEER) distance measurement. We labeled the MBD after introducing a single cysteine at a permissive site (A117C; Figure S7) with methanethiosulfonate spin label (MTSL; Figure S8) and obtained the spin-labeled DNA duplex from an oligonucleotide that contained a single phosphorothioate group that was reacted with freshly iodinated 1-oxyl-2,2,5,5-tetramethyl-3(methanesulfonyloxymethyl)-pyrroline (Figure 5c). As expected,^32^ the distance distributions (Figures S9 and S10) for the wt MeCP2MBD-DNA (mC/mC) complex indicated two (differently populated) binding orientations (Figure 5c). In contrast, the MeCP2 T/A/Y/N mutant bound to the duplex with an asymmetric hmC/mC CpG dyad predominantly in one specific orientation (Figure S11) that placed N134 closer to the hmC and the unsubstituted Y134 closer to the mC (Figure S12). This data further support a positive recognition mode in which T/A/Y/N is bound via two defined residue sets for the different 5-substituents.

**Figure 6:**
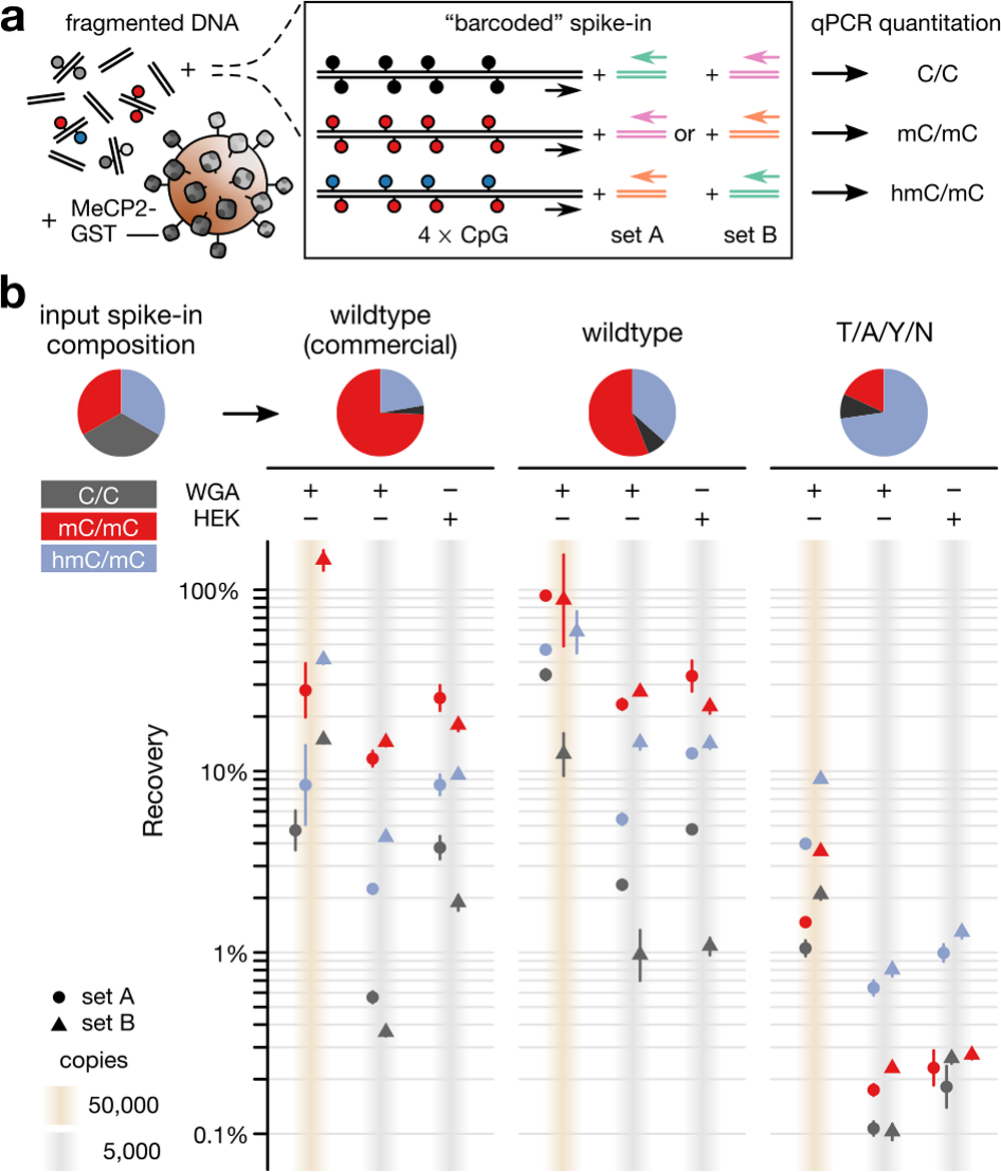
Selective and sensitive enrichment of hmC/mC CpGs from genomic DNA backgrounds by MeCP2 T/A/Y/N. (a) Assay design using an equimolar spike-in of three modified 79-mer DNA duplexes with four modified or unmodified CpGs. Measurements were conducted with two different combinations of barcode sets. (b) Recovery of the differentially modified target DNA duplexes normalized to the input; error bars are means of duplicate enrichments and duplicate qPCR measurements for each set. Pie diagrams show relative distribution of targets in input and the different enrichments for 5000 copies and HEK293T gDNA background.

To test whether MeCP2 T/A/Y/N would be useful for the analysis of hmC/mC-containing DNA fragments in genomic DNA backgrounds, we next conducted affinity enrichment experiments. We diluted a mixture of 79-mer DNA duplexes containing 4 CpGs (reflecting typically recovered, local CpG densities)^33^ bearing either C/C, mC/mC, or hmC/mC into human genomic DNA (gDNA) at a representation of 50 000 genome copies each (Figure 6a). This introduces a number of hmC/mC-modified CpGs that corresponds to 0.7% of all CpGs in the sample and is in the lower range of cellular hmC levels.^8^ We equipped the spike-ins with unique primer binding sites (“barcodes”) so that their relative abundance after the enrichment could be measured by quantitative PCR (qPCR). We constructed C-terminal GST fusion proteins^34^ of the wt and the T/A/Y/N MeCP2 MBD and employed them in a commercial “MethylCap” assay.^35^ Initially, we used modification-free, whole-genome amplified human gDNA as background. Here, the wt MBD showed a 2.5- to 3.5-fold enrichment of mC/mC over hmC/mC, which was similar to the commercial wt MBD-GST fusion of MeCP2 of the MethylCap kit (Figure 6b). In contrast, the T/A/Y/N MeCP2 showed a 3.5- to 4.5-fold enrichment of hmC/mC over mC/mC or C/C (Figures 6b and S13). Importantly, we obtained similar results using the same targets with swapped barcodes, excluding the possibility of barcode-dependent bias. Moreover, we also obtained similar results for samples containing only 5000 copies of the spike-ins using either the same or a naturally methylated gDNA background obtained from HEK293T cells (Figure 6b). We could further confirm the hmC/mC selectivity of T/A/Y/N in competitive enrichments with the alternative off-target combinations C/mC and hmC/C (Figure S14).

Overall, the enrichments thus confirmed the higher selectivity of the T/A/Y/N mutant compared to wt MeCP2 that we observed in EMSA in respect to their respective on- and off-target CpG dyads mC/mC and hmC/mC (Figure 4c). However, we also observed a lower total target recovery for the T/A/Y/N mutant as compared to wt MeCP2 in the enrichments. Although a high recovery rate is not essential for this method and we observe selective enrichment even at low target copy numbers, we envision that this aspect could be further improved. Given the similar affinity of both probes under our EMSA conditions (Figure 4b–d), the lower recovery may be explained by the buffer conditions of the MethylCap protocol that have been optimized for wt MeCP2 but not for the T/A/Y/N mutant. Taken together, this demonstrates that our novel probe can enrich hmC/mC-containing targets with high sensitivity and selectivity even in the presence of prevalent off-target combinations in the same sequence context and in the presence of a large number of off-target mC/mC CpGs in gDNA.

## Conclusion

We developed a rapid selection system for MBDs based on *E. coli* cell-surface display and employed it to evolve the first affinity probe for hmC/mC CpG dyads. This probe positively and selectively recognizes hmC/mC dyads out of all 15 possible modified CpG dyads and rivals the selectivity of the naturally evolved wt MBD for mC/mC. This hints at an evolutionary plasticity of the MeCP2 MBD for the selective recognition of noncognate CpG dyad modifications that may be exploitable for targeting other strand-symmetric or strand-asymmetric combinations. We chose hmC/mC as initial target for directed evolution because of its role as first oxidation product that TETs generate from mC/mC CpGs and because of its expected high relative abundance among dyads containing oxidized mC derivatives. In our affinity enrichment experiments, we observed a higher selectivity for T/A/Y/N MeCP2 compared to wt MeCP2 with respect to their opposing on- and off-target CpG dyads mC/mC and hmC/mC. However, the overall selectivity of both wt MBD and T/A/Y/N without further evolutionary optimization is in a range where successful applications at an acceptable false discovery rate will depend on abundance and density of modified CpGs. A reasonable genome specimen for mapping of hmC/mC dyads might thus be embryonic stem cells or neuronal tissue with inherently high (and in the latter case also stable) hmC levels.^26,37,38^ We however also noted a significant enrichment of hmC-containing off-target CpG modifications for the wt MeCP2, with consequences for expected false discovery rates in these cell types for the commonly used MethylCap assay. In conclusion, given the versatile use of MBDs for the analysis of mC in vitro and in vivo^16,39−41^ as well as the observed selectivity of our new probe, we anticipate that it will serve as a useful tool for studying the functions of hmC/mC marks in diverse aspects of chromatin biology.

## Supporting Information Available

Additional data, methods and materials for interaction analyses, EPR and NMR measurements, and enrichment experiments (PDF)
