# Supplementary material for: Evolved DNA Duplex Readers for Strand-Asymmetrically Modified 5-Hydroxymethylcytosine/5-Methylcytosine CpG Dyads
Source: J Am Chem Soc. 2022 Feb 14;144(7):2987–93. doi: 10.1021/jacs.1c10678 (PMC8874921; doi:10.1021/jacs.1c10678)
Supplement: Supplementary file 1 — ja1c10678_si_001.pdf [file ja1c10678_si_001.pdf]

Supporting Online Information

Evolved DNA Duplex Readers for Strand-Asymmetrically Modified 5-Hydroxymethylcytosine/5-Methylcytosine CpG Dyads

Benjamin C. Buchmuller[a], Jessica Dröden[b], Himanshu Singh[a], Shubhendu Palei[a], Malte Drescher[b], Rasmus Linser*[a] and Daniel Summerer*[a]

[a]Faculty of Chemistry and Chemical Biology, TU Dortmund University, Otto-Hahn-Straße 4a, 44227 Dortmund, Germany

[b]Department of Chemistry and Konstanz Research School of Chemical Biology, University of Konstanz, Universitätsstraße 10, 78457 Konstanz, Germany

Table of Contents

Table of Contents 2

Supplementary Figures 3

Figure S1. Validation of FACS screening conditions. 3

Figure S2. Separation efficiency on FACS. 4

Figure S3. Reproducibility of EMSA on FACS. 5

Figure S4. Genotype enrichment after FACS selection of an MeCP2 mutant library. 6

Figure S5. Binding affinity of selected mutants. 7

Figure S6. Binding affinity in natural sequence contexts. 8

Figure S7. Binding selectivity of MeCP2[A117C] and MeCP2[K109T/A117C/V122A/S134N]. 9

Figure S8. ESI-MS spectrum of MTSL-labeled MeCP2[K109T/A117C/V122A/S134N]. 9

Figure S9. DEER data of MeCP2 bound to DNA with a modified CpG. 10

Figure S10. Control experiment with wildtype MeCP2 (without A117C) bound to mC/mC. 11

Figure S11. Quantification of binding orientation. 11

Figure S12. Model of MeCP2 T/A/Y/N mutant in complex with hmC/mC-modified CpGs. 12

Figure S13. Enrichment from complex mixtures (C/C, mC/mC, hmC/mC). 13

Figure S14. Enrichment from complex mixtures (hmC/C, C/mC, hmC/mC). 14

Figure S15. Entry vector for bacterial surface-display. 15

Figure S16. Assembly gaps for library creation. 15

Supplementary Tables 17

Table S1. Plasmids. 17

Table S2. Oligodeoxynucleotides (ODNs) for cloning. 18

Table S3. ODNs for FACS. 19

Table S4. ODNs for EMSA and EPR spectroscopy. 19

Table S5. ODNs used in the qPCR assay. 20

Table S6. ODN pairs for FACS and EMSA. 21

Table S7. Fit statistics for oligo-dA/dT context. 22

Table S8. Fit statistics for natural contexts. 24

Methods 25

References 36

Supplementary Figures

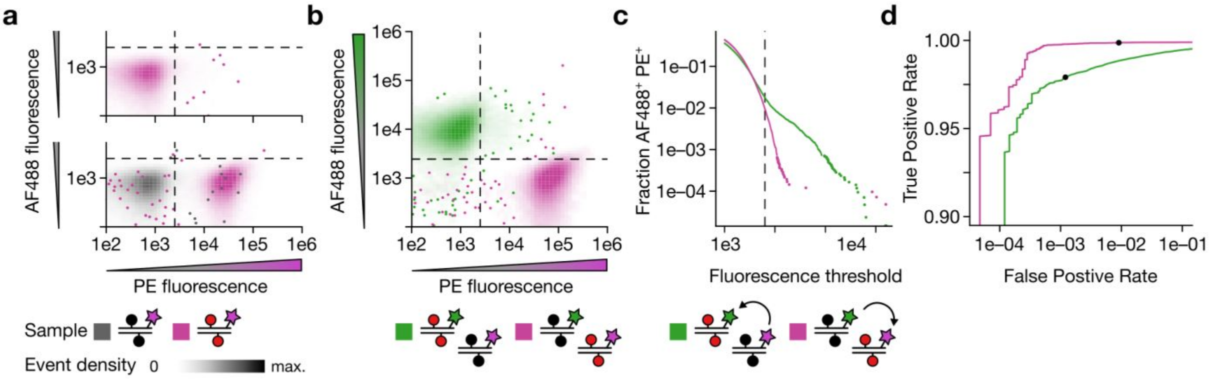

Figure S1. Validation of FACS screening conditions. (a) Overlay of FACS scatter plots (compensated fluorescence intensities according to the instrument's manufacturer) from surface-displayed "empty" AIDA (pBeB1383, top panel) or MBD2 (pBeB1567, lower panel) after staining with phycoerythrin (PE)-labeled DNA duplexes that contain either a single C/C (i.e., unmodified; gray) or a single mC/mC CpG dyad (i.e., fully methylated; magenta; one-color FACS DNA-binding assay; both shown in Figure 2d, panel 1 and 2 from the left). **(b)** Double-staining of the MBD2 displaying cells (pBeB1567) with an equimolar mixture of C/C and mC/mC duplexes in which one is labelled with PE, the other with AF488 (green: mC/mC-AF488 and C/C-PE; magenta: mC/mC-PE and C/C-PE; two-color FACS DNA-binding assay; Figure 2d, panel 3 and 4 from the left). The sparsest 0.1% of events in *a* or *b* are indicated as dots; Dashed lines show the minimum thresholds for gating. **(c)** Fraction of events above both gating thresholds (dashed line as in *b*), i.e., "unexpected" PE-positive events in the double-staining of mC/mC-AF488 and C/C-PE and vice versa. In practical terms of the screening, this rate relates to the likeliness of detecting a non-specific binder, i.e., the false positive rate of the assay procedure prior to the actual measurement on the instrument. Dots indicate threshold levels populated with less than 30 double-positive events. **(d)** True positive rate (sensitivity) and false positive rate (1 – specificity) at various gate settings calculated from *b* for each fluorophore combination (black dots are thresholds shown in *b*). Here, false positives are events that originate from detection of a (supposedly different) duplex with similar affinity as the duplex in question, but labeled with a different fluorophore, i.e., "red

dots in green gate”. The false positive rate is a worst-case lower bound since in reality, the gating can be constraint in the direction of the second fluorophore as well.

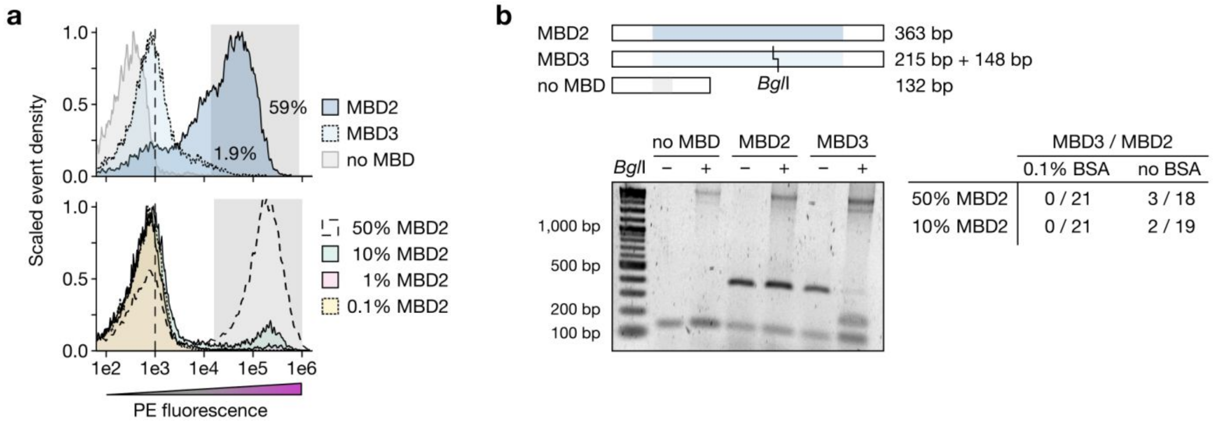

Figure S2. Separation efficiency on FACS. (a) Single-color staining with a phycoerythrin (PE)-labeled mC/mC CpG-duplexes; Upper panel with surface-displayed "empty" AIDA (pBeB1383; "no MBD"), surface-displayed MBD2 (high mC/mC-affinity MBD[1]; pBeB1567) or MBD3 (low mC/mC-affinity MBD[1]; pBeB1568); Lower panel with a mix of MBD2- and MBD3-displaying cells; mixed before induction of the surface-display based on OD600 measurements. Shaded areas indicate gate used for sorting in *b*. **(b)** Single-clones sorted from mixtures of surface-displayed MBD2 and MBD3 onto agar plates (LB with antibiotic) and analyzed for MBD3-postive clones by colony PCR and subsequent *BglI* restriction digest. Note that blocking with 0.1% BSA during resuspension of the mixed cultures in PBS reduced the number of MBD3-positive colonies which indicates unspecific probe binding in absence of BSA. BSA blocking was thus used during later selections.

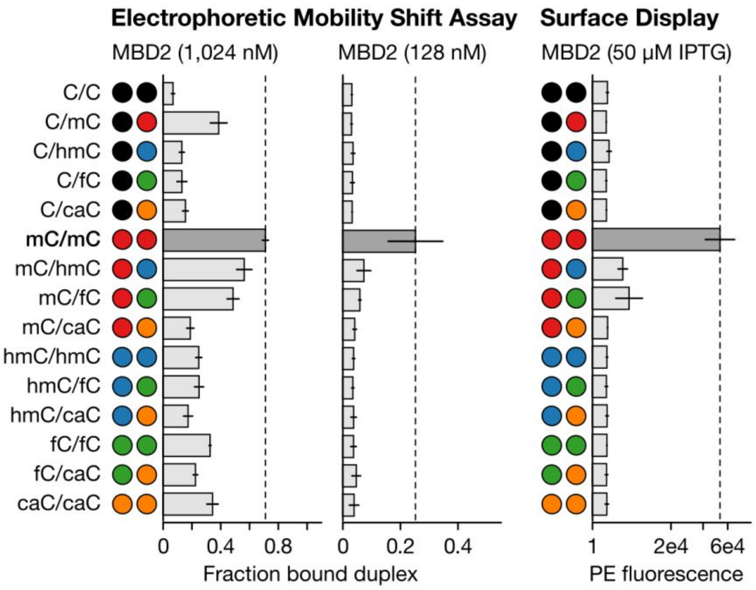

Figure S3. Reproducibility of EMSA on FACS. Fraction of bound 6-FAM-labeled DNA duplexes bearing different modified CpG dyads using electrophoretic mobility shift assay (EMSA) with recombinantly expressed MBD2 at a high and low protein concentration (reproduced from Buchmuller et al., 2020[2]) in comparison to the same duplexes (biotinylated and labeled with phycoerythrin, PE) on a single-color FACS binding assay with surface-displayed MBD2. Error bars in *a* and *b* indicate mean ± SEM of three technical replicates.

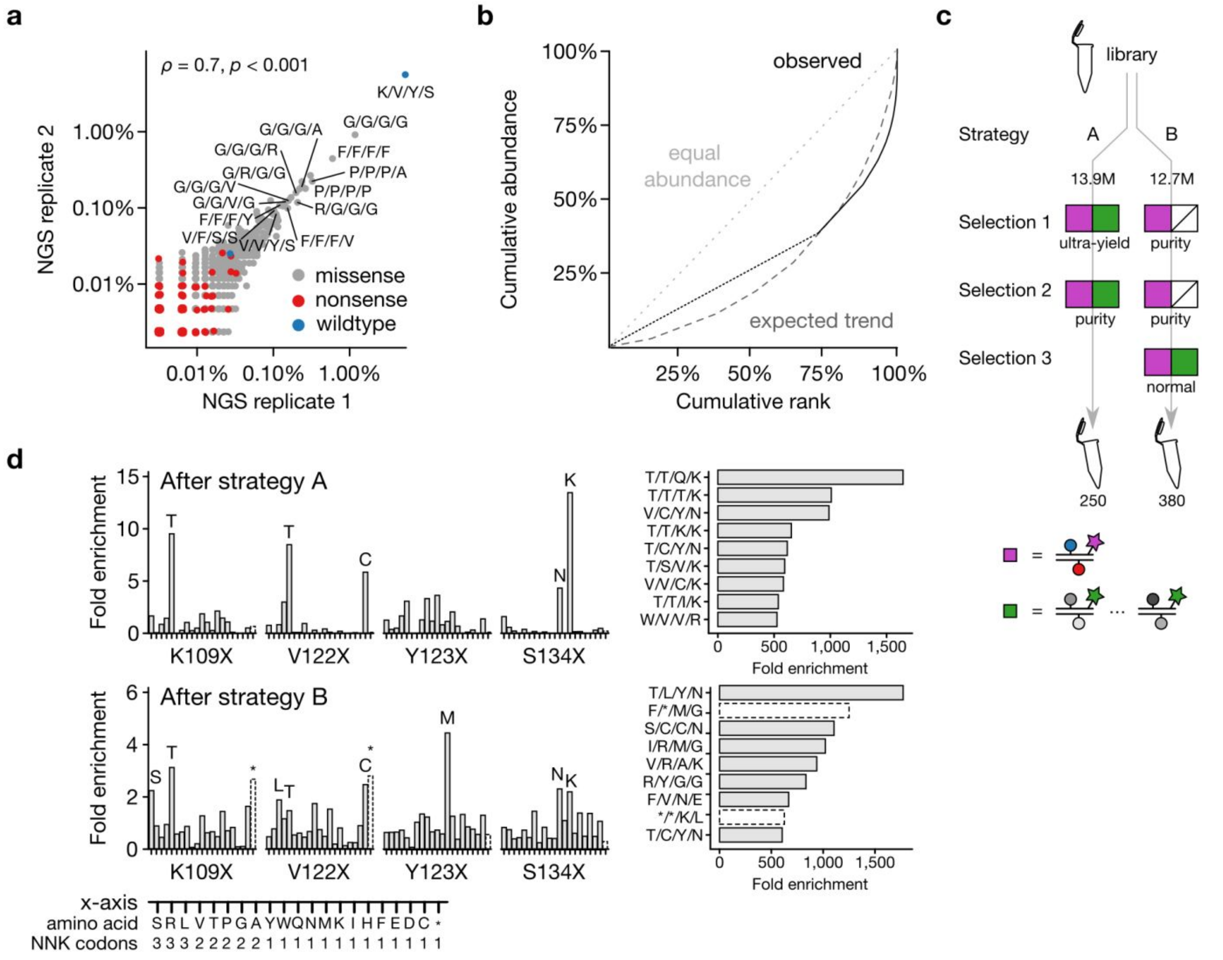

Figure S4. Genotype enrichment after FACS selection of an MeCP2 mutant library. (a) Diversity of the degenerated initial library MeCP2[K109X/V122X/Y123X/S134X] based on short-read next-generation sequencing and UMI counting within 70,000 distinct sequences (see Methods). Out of 49,300 genotypes, 6,000 were observed in both sequencing replicates (data points shown for these only), indicating >87% unique genotypes; Pearson's correlation coefficient given. The sampled genotypes coded for 33,900 distinct phenotypes with 7.8% amber (TAG) nonsense mutants (expected: 11.9%), 88.5% missense mutants (expected: 88.1%) and 3.7% wildtype MeCP2 (expected: <0.01%). The number of stop codons disallowed by the used NNK mutagenesis (TAA, TGA) was <0.09%. **(b)** Phenotypes of *a* (combined sequencing replicates) ranked by abundance compared to the cumulative ranked distribution of a theoretical NNK degenerated library with four positions and equal amounts of nucleobases (expected trend) and a trimer-based combinatorial library (equal abundance of all amino

acid combinations). Dotted section of the observed trend indicates phenotypes with 1 UMI only. **(c)** Sorting strategies employed; magenta indicates the use of a staining with hmC/mC probes labeled with phycoerythrin, green a staining with a drop-out mix labeled with AF488 (14 other probes, no hmC/mC duplex as described under Methods). Figure 3a relates to strategy A. **(d)** Amino-acid substitutions enriched after the final sorting step indicated in *c* per NNK degenerated position (left) and in combination for distinct phenotypes (right). Top panels shown partially in Figure 3b.

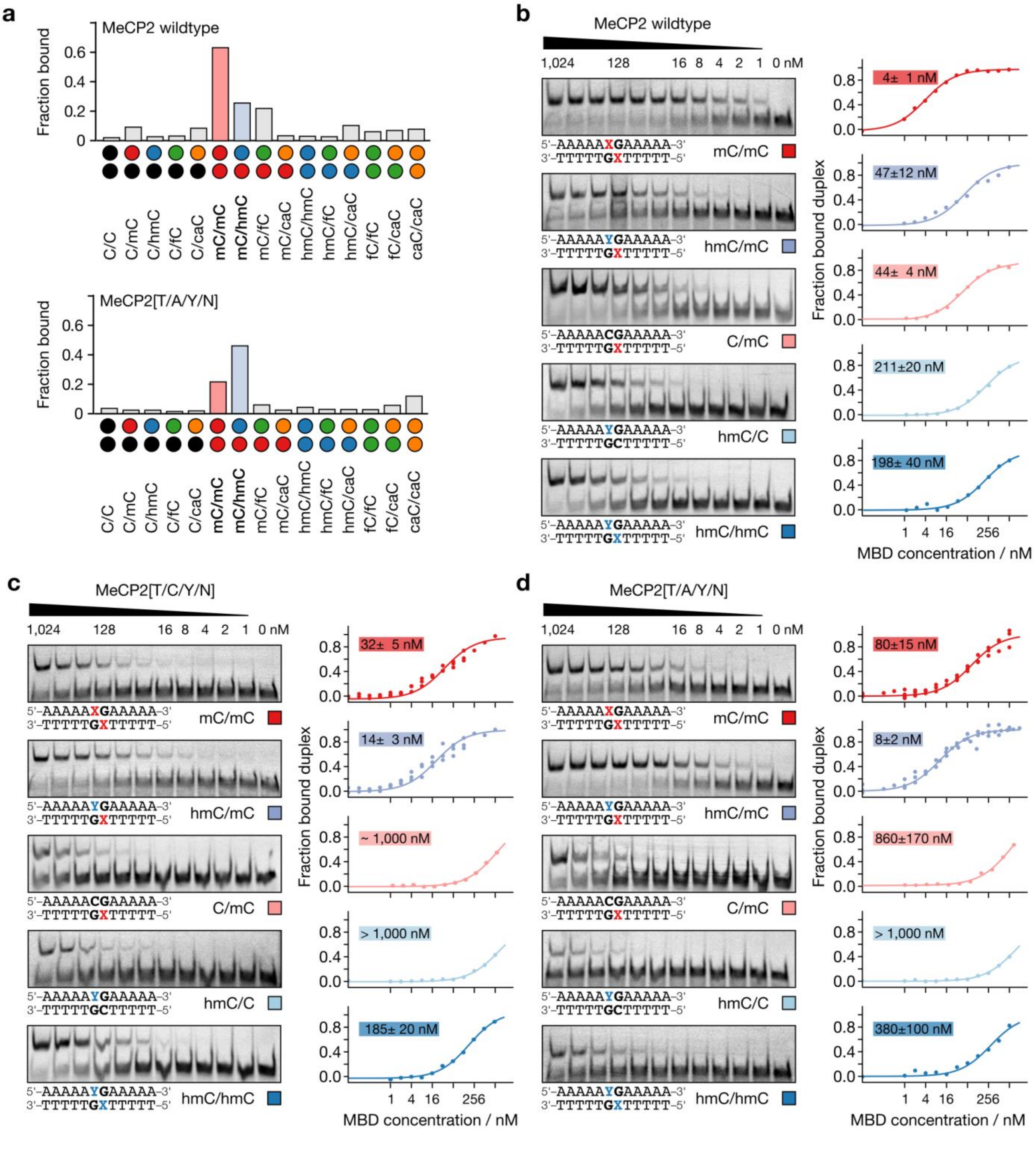

Figure S5. Binding affinity of selected mutants. (a) Electrophoretic mobility shift (EMSA) selectivity profiles of the recombinantly expressed MBD of wildtype MeCP2 and the MeCP2[K109T/V122A/S134N] at 10 nM MBD for different modified CpG dyads. **(b)** EMSA gel images of a dilution series of wildtype MeCP2 MBD at 1,024, 512, 256, 128, 64, 32, 16, 8, 4, 2, 1 and 0 nM with 2 nM of the labeled

DNA duplexes containing the indicated 2'-deoxycytidine modifications in an oligo-dA/dT context. **(c)** Same as *b* for MeCP2[K109T/V122C/S134N], **(d)** MeCP2[K109T/V122A/S134N]. Fitted isotherms are reproduced in Figure 4c.

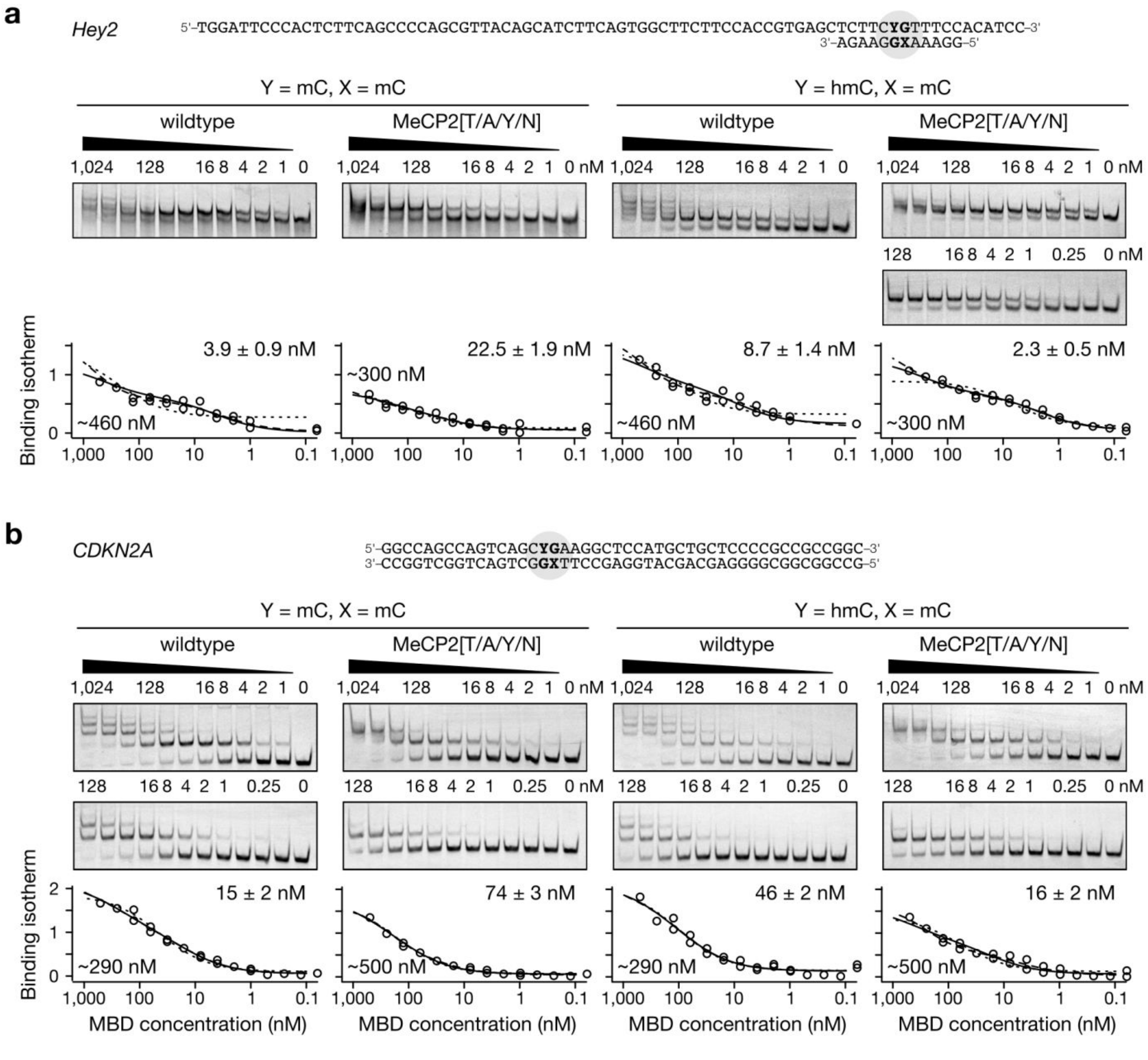

Figure S6. Binding affinity in natural sequence contexts. Electrophoretic mobility shift (EMSA) gel images with fluorescently labeled DNA duplexes containing a single modified CpG for wildtype MeCP2 and the MeCP2[K109T/V122A/S134N] mutant. Since an oligo-dA/dT duplex was employed as "dark" competitor, this assay also reveals binding of non-CpG sequences and unmodified CpGs. **(a)** For an intronic sequence of the *Hey2* ortholog in the zebrafish *Danio rerio* (chr:20:39,589,641-719;

danRer11). **(b)** With a single modified CpG in the sequence context of the first exon of CDKN2A (chr9:21,974,777–822; hg38). First (dotted), second (solid; estimates reported in Table S8 and Figure 4d) and third (dashed) order polynomial fits, macroscopic binding constants, observed data (points).

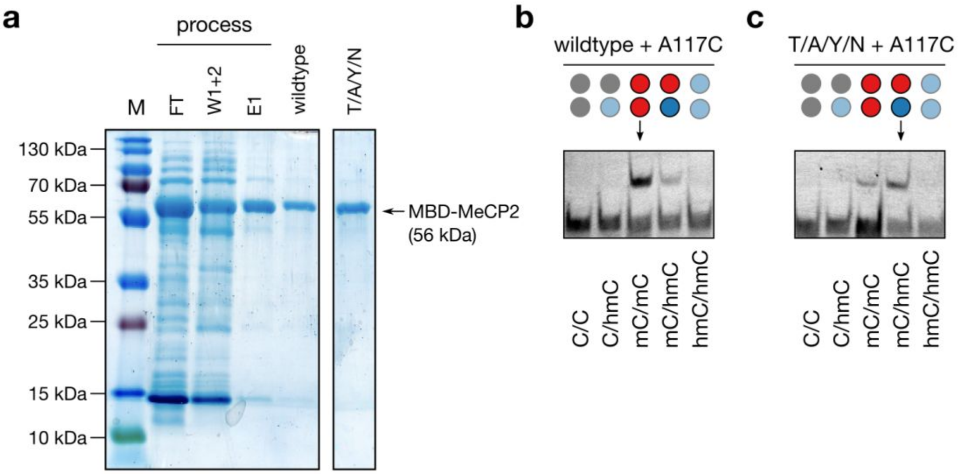

Figure S7. Binding selectivity of MeCP2[A117C] and MeCP2[K109T/A117C/V122A/S134N]. (a) Expression and purification of A117C variants; flowthrough (FT), washing (W1+2) and elution for wildtype and T/A/Y/N mutant MeCP2. **(b)** Binding selectivity at 256 nM of the cysteine wildtype variant and (c) of the A117C T/AY/N mutant used for EPR spectroscopy.

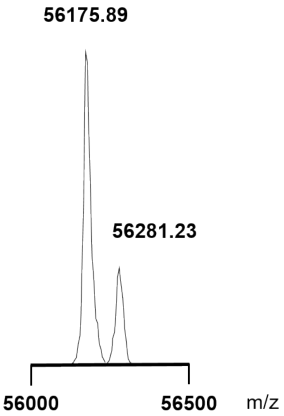

Figure S8. ESI-MS spectrum of MTSL-labeled MeCP2[K109T/A117C/V122A/S134N]. Highest peak corresponds to successfully labeled protein (calculated m/z: 56,175.58; detected m/z: 56,175.89). The other peak (m/z = 56,281.23) exhibits a m/z ratio that is 105.64 u larger than found for the highest peak, which arises from PEG2 adding to the protein while LC/MS analysis and is introduced during the measurement.

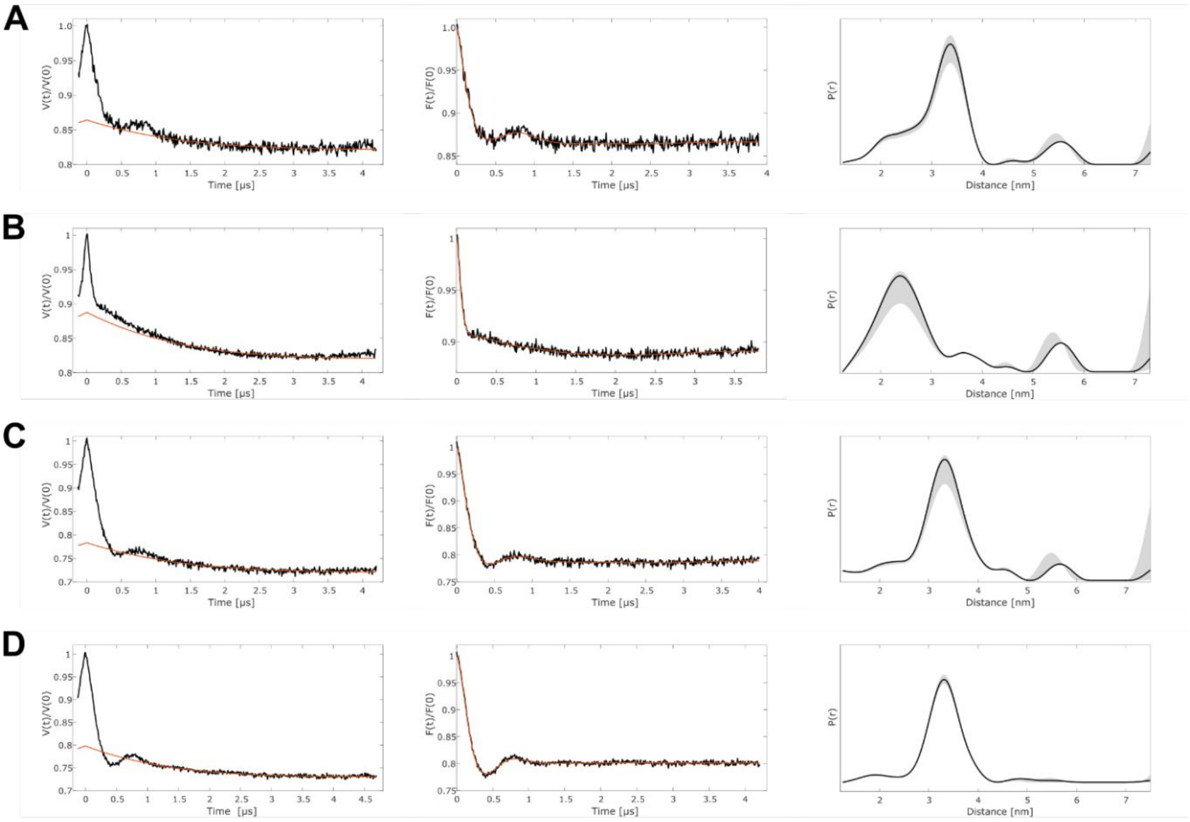

Figure S9. DEER data of MeCP2 bound to DNA with a modified CpG. Raw data (left), form factor (middle) and resulting distance distribution after Tikhonov regularization (right). Grey shaded areas present uncertainties after data validation. **A** wildtype MeCP2[A117C] bound to mC/mC-dT* DNA duplex (o2967/o4328). **B** wildtype MeCP2[A117C] and mC/mC-dA* duplex (o4329/o2909). **C** The T/A/Y/N MeCP2 mutant MeCP2[K109T/A117C/V122A/S134N] bound to mC/mC-dT* duplex or **D** interacting with mC/hmC-dT* DNA duplex (o3115/o4328). Distances between 5 and 6 nm found in both measurements were reviewed in a control experiment using wt-MeCP2 bound to singly labeled DNA (mC/mC-dT*), see Figure S10.

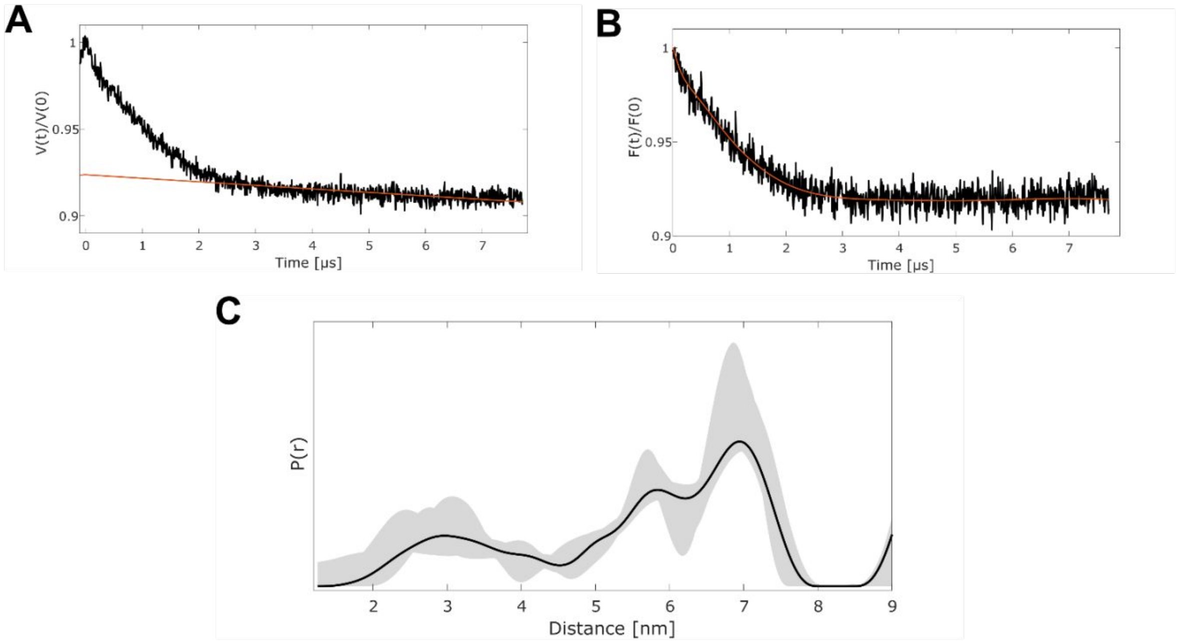

Figure S10. Control experiment with wildtype MeCP2 (without A117C) bound to mC/mC. A Raw data, **B** form factor and **C** distance distribution after Tikhonov regularization using the L-curve criterion. Grey shaded area presents uncertainty after data validation. The presence of longer distances in the range of 5 to 7.5 nm arising from inter-molecular interactions. This behavior has been reported to originate from end-to-end stacking of short, double-stranded oligonucleotides.[3,4]

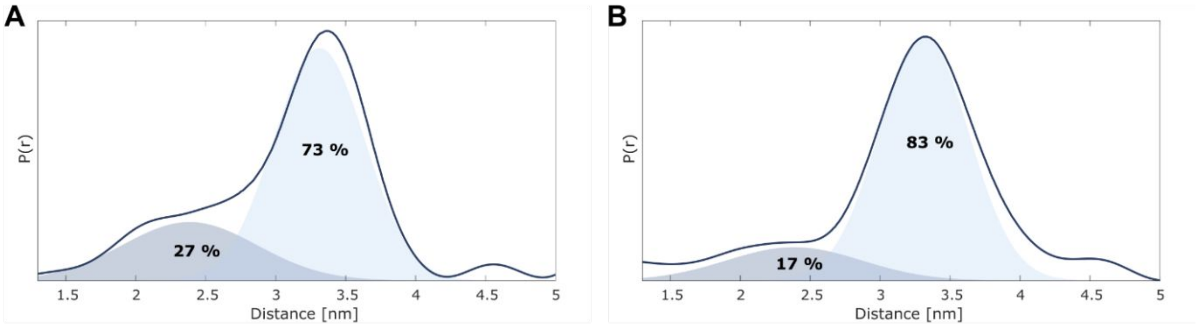

Figure S11. Quantification of binding orientation. A “Wildtype” MeCP2[A117C] and **B** the T/A/Y/N mutant MeCP2[K109T/A117C/V122A/S134N] bound to mC/mC-dT* DNA. Distance distribution fitted with two Gaussians (blue shaded areas) and area under each curve to calculate the percentage of each orientation.

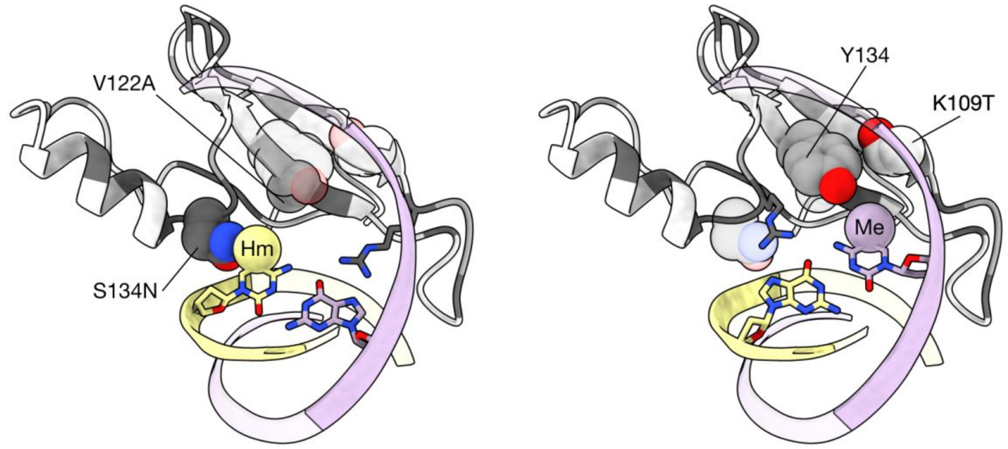

Figure S12. Model of MeCP2 T/A/Y/N mutant in complex with hmC/mC-modified CpGs. Illustrative model based on the crystal structure of wildtype MeCP2 (PDB: 3c2i) with the K109T, V122A and S134N substitutions (residues swapped with ChimeraX) highlighting the two distinct sets of amino acid residues in vicinity to each modified DNA nucleobase as suggested by EPR spin-labeling and DEER measurements (Figure 5c).

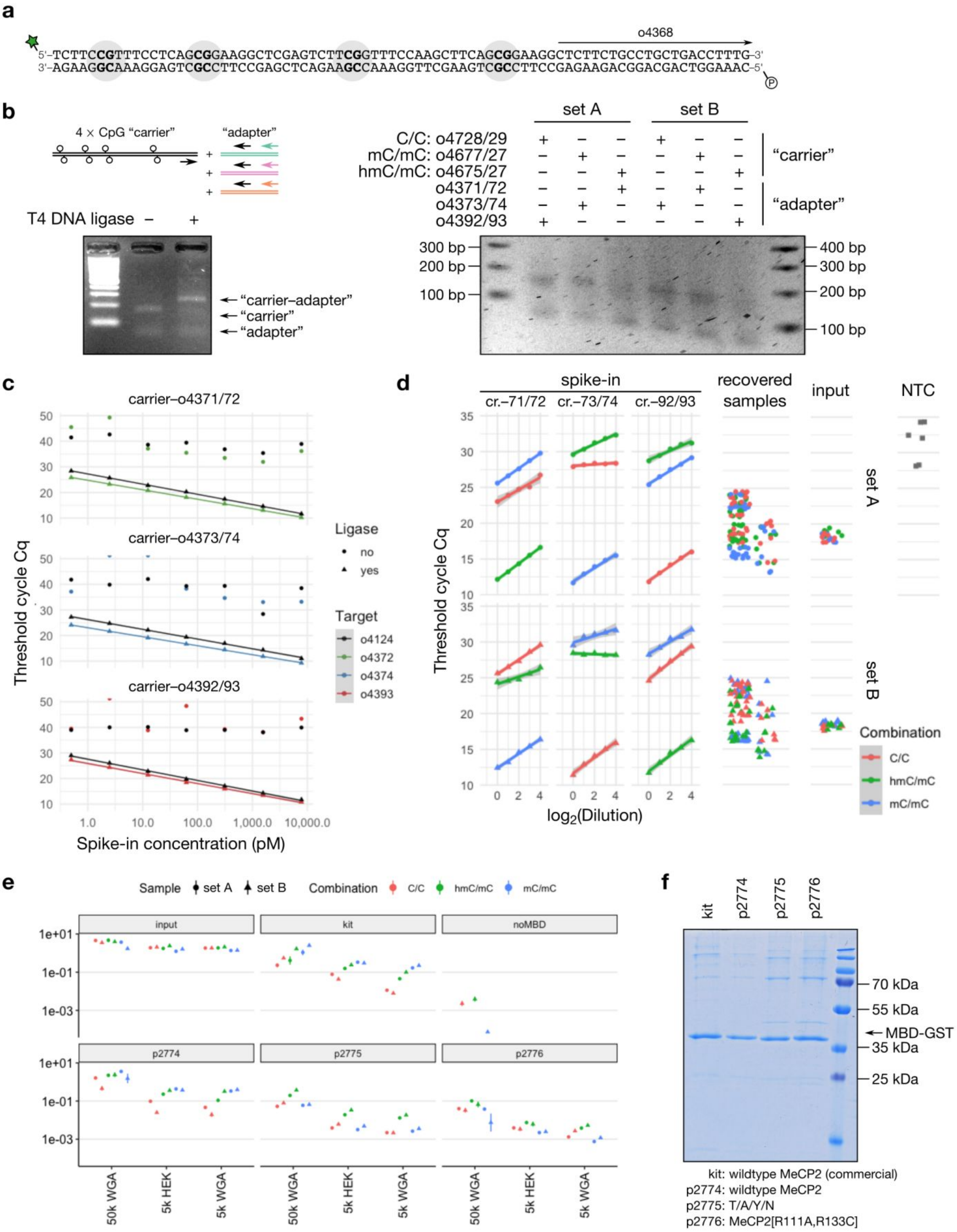

Figure S13. Enrichment from complex mixtures (C/C, mC/mC, hmC/mC). (a) Sequence of a (unmodified) "carrier" with the four modified CpG dyads highlighted. **(b)** Representative agarose gel images of the purified carrier–qPCR adapter ligation products (Methods) for a longer sample carrier (left, not used in this study) and the carriers used in this study (right). **(c)** Even in presence of remaining "adapter" after purification, the concentration of the carrier–adapter junctions can be reliably

quantified using the common or the specific primer binding site. **(d)** Standard curve from a dilution series of spike-ins and raw Cq values of samples evaluated in this study. Note that all samples have Cq values lower than those observed as “cross-talk” in the dilution series. **(e)** Extrapolated recovery prior to normalization to the input shown in Figure 6b, including recovery with a non-binding MeCP2 (R111A, R133C mutation; pBeB2776) under the same conditions. Note that the T/A/Y/N mutant (pBeB2775) recovers the hmC/mC spike-in significantly above this unspecific level. **(f)** Recovery of the indicated GST-MBD proteins from the GSH beads after enrichment (50,000 copies, WGA of *e* shown as representative example).

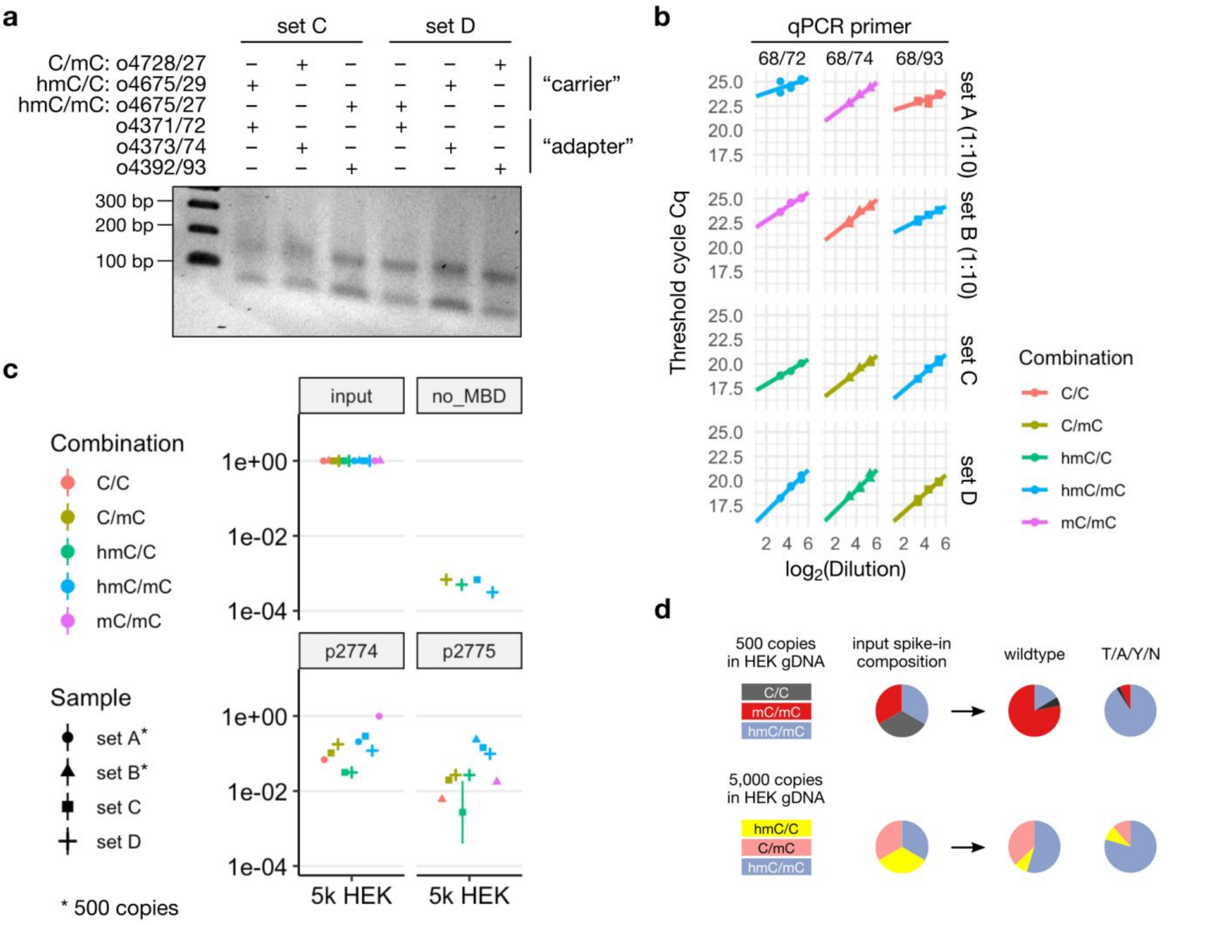

Figure S14. Enrichment from complex mixtures (hmC/C, C/mC, hmC/mC). (a) Purified carrier–qPCR adapter ligation products for two other sets of carriers with combinations of modified CpG dyads different from the ones in Figure S13. **(b)** Dilution series and quantitation of the mixed carrier–adapters including a 1:10 dilution of “set A” and “set B” of Figure S13 as control. (c) Extrapolated recovery prior to normalization to the input of four enrichments per protein (wildtype MeCP2, pBeB2774; T/A/Y/N mutant, pBeB2775; no MBD control), one for each “Sample” (set A, B, C, D) containing either 500 copies (set A, B) or 5,000 copies (set C, D; comparable to Figure S13) with different mixtures in different barcode combinations. To facilitate comparison, values for each Sample are combined in each panel; Error bars are qPCR duplicates. **(d)** Inferred composition (averages of set A and B or C and D respectively) before and after the enrichments shown in *c*.

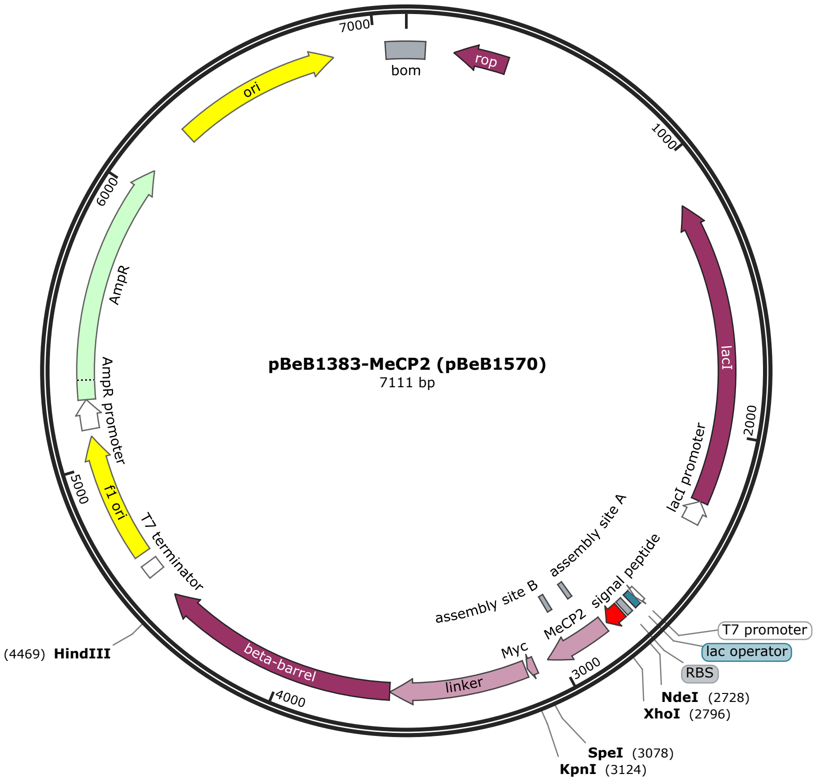

Figure S15. Entry vector for bacterial surface-display. Example with sub-cloned MeCP2 wildtype in the *Spe*I/*Xho*I cloning site. Map created with SnapGene software (from Insightful Science; available at www.snapgene.com).

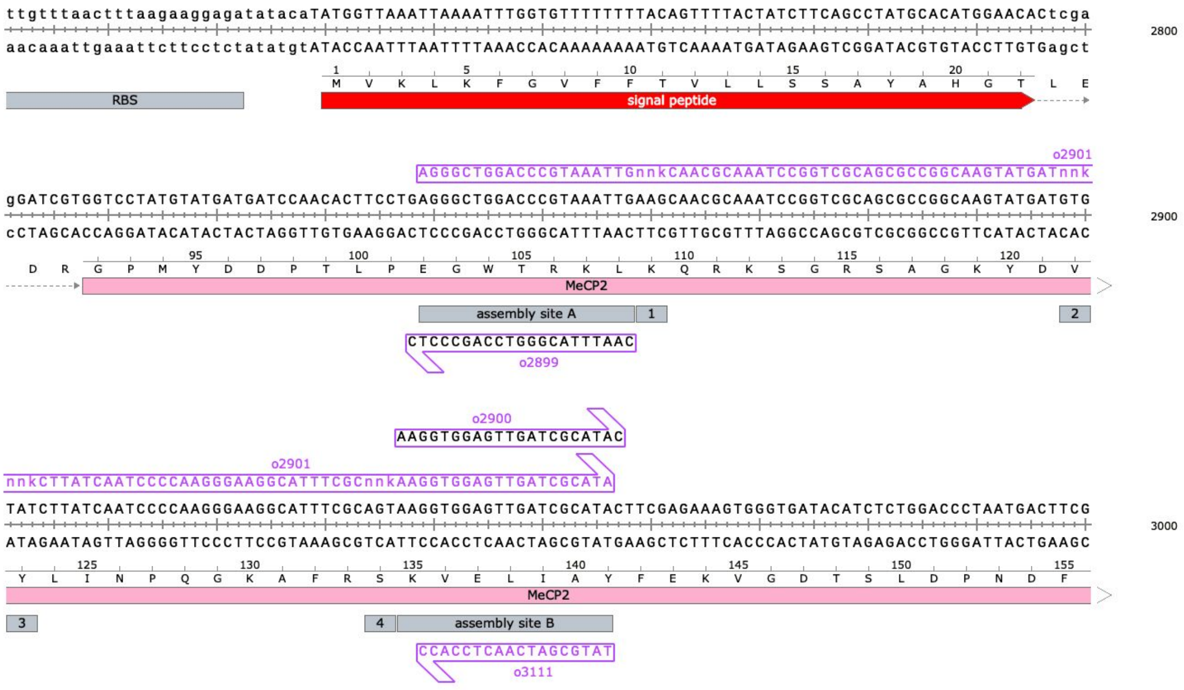

Figure S16. Assembly gaps for library creation. Primer binding sites for backbone linearization and generation of the NNK codon-degenerated insert (Methods). Map created with SnapGene software (from Insightful Science; available at www.snapgene.com).

Supplementary Tables

Table S1. Plasmids.

**pBeB1383**	pET(21d)-CtxB_SPase-(MCS)-[c-myc]-AIDA_linker_barrel (AmpR)
**pBeB1567**	pBeB1383-hMBD2[146–225]
**pBeB1568**	pBeB1383-hMBD3[2–81]
**pBeB1570**	pBeB1383-hMeCP2[90–181]
**pJeJ2370**	pBeB1383-hMeCP2[90–181][R111A,R133A]
**pBeB1727**	pBeB1383-hMeCP2[90–181][K109X/V122X/V123X/S134X]
**pBeB1778**	pBeB1383-hMeCP2[90–181][K109T/V122T/Y123T/S134K]
**pBeB1779**	pBeB1383-hMeCP2[90–181][K109T/V122A/S134N]
**pKaB2620**	pBeB1383-hMeCP2[90–181][K109T/V122C/S134N]
**pBeB2638**	pBeB1383-hMeCP2[90–181][K109T/V122L/S134N]
**pBeB2644**	pBeB1383-hMeCP2[90–181][K109T/V122T/Y123Q/S134K]
**pBeB1379**	pET(21d)-[FLAG]-MBP-[Factor Xa]-[TEV]-(MCS)-6xHis (AmpR)
**pBeB1785**	pET(21d)-[FLAG]-MBP-[Factor Xa]-[TEV]-(MCS)-6xHis (CmR)
**pBeB1385**	pBeB1379-hMBD2[146–225]-6xHis
**pBeB1386**	pBeB1379-hMBD3[2–81]-8xHis
**pBeB1388**	pBeB1379-hMeCP2[90–181]-7xHis
**pJeJ2091**	pBeB1379-hMeCP2[90–181][R111A,R133A]
**pBeB1856**	pBeB1785-hMeCP2[90–181][K109T/V122T/Y123T/S134K]
**pBeB1859**	pBeB1785-hMeCP2[90–181][K109T/V122A/S134N]
**pBeB2526**	pBeB1388-hMeCP2[90–181][K109T/V122C/S134N]
**pBeB2527**	pBeB1388-hMeCP2[90–181][K109T/V122T/Y123Q/S134K]
**pBeB2573**	pBeB1388-hMeCP2[90–181][A117C] (AmpR)
**pBeB2577**	pBeB1785-hMeCP2[90–181][K109T/A117C/V122A/S134N] (CmR)
**pBeB2774**	pBeB1379-hMeCP2[90–181]-GST-6xHis (AmpR)

**pBeB2775**	pBeB1785-hMeCP2[90–181][K109T/V122A/S134N]-GST-6xHis (CmR)
**pBeB2776**	pBeB1379-hMeCP2[90–181][R111A,R133A]-GST-6xHis (AmpR)

Table S2. Oligodeoxynucleotides (ODNs) for cloning.

o2899	CAATTTACGGGTCCAGCCCTC
o2900	AAGGTGGAGTTGATCGCATAC
o2901	AGGGCTGGACCCGTAAATTGnnkCAACGCAAATCCGGTCGCAGCGCCGGCAAGTATGATnnknnkCTTATCAAT<br>C CCCAAGGGAAGGCATTTCGCnnkAAGGTGGAGTTGATCGCATA
o2912	ATGGTTAAATTAAAATTTGGTGTTTTTTTTACAGTTTTACTATCTTCAGCCTATGCACATGGAACACtcgaggtagc<br>ag tgactagtcaccaccaccaccaccacGGTACCCTTAATCCTACAAAAGAAAGTGCAGGTAATACTCTTAC<br>CGTGTCAAATTATACTGGGACACCGGGAAGTGTTATTTCTCTTGGTGGTGTGCTTGAAGGAGATAATTCACTT<br>ACGGACCGTCTGGTGGTGAAAGGTAATACCTCTGGTCAAAGTGACATCGTTTACGTCAATGAAGATGGCAGTG<br>GTGGTCAGACGAGAGATGGTATTAACATTATTTCTGTAGAGGGAAATTCTGATGCAGAATTTTCTCTGAAGAA<br>CCGCGTAGTTGCCGGAGCTTATGATTACACACTGCAGAAAGGAAACGAGAGTGGGACAGATAATAAGGGATG<br>GTATTTAACCAGTCATCTTCCCACATCTGATACCCGGCAATACAGACCGGAGAACGGAAGTTATGCTACCAAT<br>ATGACACTGGCTAACTCACTGTTCCTCATGGATTTGAATGAGCGTAAGCAATTCAGGGCAATGAGTGATAATA<br>CACAGCCTGAATCTGCATCCGTGTGGATGAGGATTACTGGAGGAAGAAGCTCTGGTAAACTTAATGACGGGC<br>AAAATAAAACAACAACCAATCAGTTTATCAATCAGCTCGGGGGGGATATTTACAAATTCCATGCTGAACAACT<br>GGGTGATTTTACCTTAGGGATTATGGGAGGATACGCGAATGCAAAAGGTAAAACGATAAATTACACGAGCAA<br>CAAAGCTGCCAGAAACACACTGGATGGTTATTCTGTCGGGGTATATGGTACGTGGTATCAGAATGGGGAAAA<br>TGCAACAGGGCTCTTTGCTGAAACTTGGATGCAATATAACTGGTTTAATGCCTCGGTGAAAGGTGACGGACTG<br>GAAGAAGAAAAATATAATCTGAATGGTTTAACCGCTTCTGCAGGTGGGGGATATAACCTGAATGTGCACACAT<br>GGACATCACCTGAAGGAATAACAGGTGAATTTTGGTTGCAGCCTCATTTGCAGGCTGTCTGGATGGGGGTTAC<br>ACCGGATACACACCAGGAGGATAACGGAACGGTGGTGCAGGGAGCAGGGAAAAATAACATTCAGACAAAAG<br>CAGGTATTCGTGCATCCTGGAAGGTGAAAAGCACCCTGGATAAGGATACCGGGCGGGAGTTCAGTCCGTATA<br>TAGAGGCAAACTGGATTCATAACACGCATGAATTTGGTGTTAAAATGAGTGATGACAGCCAGTTGTTGTCAGG<br>TAGCCGAAATCAGGGAGAGATAAAGACAGGTATTGAAGGGGTGATTACTCAAAACTTGTCAGTGAATGGCGG<br>AGTCGCATATCAGGCAGGAGGTCACGGGAGCAATGCCATCTCGGGAGCACTGGGGATAAAATACAGCTTCTG<br>A
o3004	GGATGCATATGGTTAAATTAAAATTTGGTGTTTTTTTTAC
o3005	CGATCGTCGACAAGCTTCAGAAGCTGTATTTTATCC
o3111	TATGCGATCAACTCCACC
o3994	GACCCGTAAATTGAAGCAAGCCAAATCCGGTCGCAGC
o3995	CGCTGCGACCGGATTTGGCTTGCTTCAATTTACGGGTC
o3996	CCCCAAGGGAAGGCATTTGCCAGTAAGGTGGAGTTG
o3997	CAACTCCACCTTACTGGCAAATGCCTTCCCTTGGGG
o4053	AGGGCTGGACCCGTAAATTgacCCAACGCAAATCCGGTCGCAGCGCCGGCAAGTATGATtgctatCTTATCAATC<br>CCCAAGGGAAGGCATTTCGCaacAAGGTGGAGTTGATCGCATA
o4054	AGGGCTGGACCCGTAAATTgacCCAACGCAAATCCGGTCGCAGCGCCGGCAAGTATGATacccagCTTATCAATC<br>CCCAAGGGAAGGCATTTCGCaaaAAGGTGGAGTTGATCGCATA
o4254	CAAATCCGGTCGCAGCTGCGGCAAGTATGATGTG
o4255	CACATCATACTTGCCGCAGCTGCGACCGGATTTG
o4280	CAAATCCGGTCGCAGCTGCGGCAAGTATGATGCG
o4281	CGCATCATACTTGCCGCAGCTGCGACCGGATTTG

**o4619**	GGGGATTGATAAGATACCCATCATACTTGCCGGCG
**o4620**	CGCCGGCAAGTATGATGGGTATCTTATCAATCCCC
**o4621**	GGGGATTGATAAGATACACATCATACTTGCCGGCG
**o4622**	CGCCGGCAAGTATGATGTGTATCTTATCAATCCCC
**o4623**	CCCTTGGGGATTGATAAGATATATATCATACTTGCCGGCGCTGCG
**o4624**	CGCAGCGCCGGCAAGTATGATATATATCTTATCAATCCCCAAGGG
**o4625**	CCTTGGGGATTGATAAGATATAGATCATACTTGCCGGCGCTGC
**o4626**	GCAGCGCCGGCAAGTATGATCTATATCTTATCAATCCCCAAGG
**o4763**	GCCAAAATCACCCAAAGCTAGCTCCCCTATACTAGGTTATTG
**o4764**	GGTGGTGGTGGTGGTGGTGAGCCGATTTTGGAGGATGGTC

Table S3. ODNs for FACS.

**o3244**	[BtnTg]AAAAAAAAAAACGAAAAAAAAAAA	
**o3245**	[BtnTg]TTTTTTTTTTTCGTTTTTTTTTTT	
**o3081**	[BtnTg]AAAAAAAAAAAXGAAAAAAAAAAA	X = 5-methyl-2’-deoxycytidine
**o3214**	[BtnTg]TTTTTTTTTTTXGTTTTTTTTTTT	X = 5-methyl-2’-deoxycytidine
**o3211**	[BtnTg]AAAAAAAAAAAXGAAAAAAAAAAA	X = 5-hydroxymethyl-2’-deoxycytidine
**o3215**	[BtnTg]TTTTTTTTTTTXGTTTTTTTTTTT	X = 5-hydroxymethyl-2’-deoxycytidine
**o3212**	[BtnTg]AAAAAAAAAAAXGAAAAAAAAAAA	X = 5-formyl-2’-deoxycytidine
**o3216**	[BtnTg]TTTTTTTTTTTXGTTTTTTTTTTT	X = 5-formyl-2’-deoxycytidine
**o3213**	[BtnTg]AAAAAAAAAAAXGAAAAAAAAAAA	X = 5-carboxy-2’-deoxycytidine
**o3217**	[BtnTg]TTTTTTTTTTTXGTTTTTTTTTTT	X = 5-carboxy-2’-deoxycytidine

Table S4. ODNs for EMSA and EPR spectroscopy.

An asterisk in the oligonucleotide sequence indicates a 3’-5’ phosphorothioate linkeage.

**o2968**	AAAAAAAAAAAAAAAAAAAAAAAA
**o2969**	TTTTTTTTTTTTTTTTTTTTTTTT

**o2903**	AAAAAAAAAAAACGAAAAAAAAAAAA	
**o2904**	TTTTTTTTTTTTCGTTTTTTTTTTTT	
**o2906**	[6-FAM]AAAAAAAAAAAACGAAAAAAAAAAAA	
**o2967**	[6-FAM]AAAAAAAAAAAAXGAAAAAAAAAAAA	X = 5-methyl-2'-deoxycytidine
**o2909**	TTTTTTTTTTTTXGTTTTTTTTTTTT	X = 5-methyl-2'-deoxycytidine
**o3115**	[6-FAM]AAAAAAAAAAAAXGAAAAAAAAAAAA	X = 5-hydroxymethyl-2'-deoxycytidine
**o3112**	TTTTTTTTTTTTXGTTTTTTTTTTTT	X = 5-hydroxymethyl-2'-deoxycytidine
**o3116**	[6-FAM]AAAAAAAAAAAAXGAAAAAAAAAAAA	X = 5-formyl-2'-deoxycytidine
**o3113**	TTTTTTTTTTTTXGTTTTTTTTTTTT	X = 5-formyl-2'-deoxycytidine
**o3117**	[6-FAM]AAAAAAAAAAAAXGAAAAAAAAAAAA	X = 5-carboxy-2'-deoxycytidine
**o3114**	TTTTTTTTTTTTXGTTTTTTTTTTTT	X = 5-carboxy-2'-deoxycytidine
**o4328**	6-FAM]TTTTTTTT*TTTXGTTTTTTTTTTTT	X = 5-methyl-2'-deoxycytidine
**o4329**	[6-FAM]AAAAAAA*AAAAXGAAAAAAAAAAAA	X = 5-methyl-2'-deoxycytidine
**o517**	TGGATTCCCACTCTTCAGCCCCAGCGTTACAGCATCTTCAGTGGCTTCTTCCACCGTGAGCTXTTCXGTTTCCAC ATCC	X = 5-methyl-2'-deoxycytidine
**o520**	TGGATTCCCACTCTTCAGCCCCAGCGTTACAGCATCTTCAGTGGCTTCTTCCACCGTGAGCTCTTCXGTTTCCAC ATCC	X = 5-hydroxymethyl-2'-deoxycytidine
**o4379**	[6-FAM]GGAAAXGGAAGA	X = 5-methyl-2'-deoxycytidine
**o4823**	[6-FAM]GGATGTGGAAAXGGAAGAGCTCACGGTGGAAGAAGCCACTGAAGATGCTGTAACGTGGGGCTGAAGA G TGGGAATCCA	X = 5-methyl-2'-deoxycytidine
**o1592**	GGCCAGCCAGTCAGCNGAAGGCTCCATGCTGCTCCCCGCCGCCGGC	X = 5-methyl-2'-deoxycytidine
**o1593**	GGCCAGCCAGTCAGCXGAAGGCTCCATGCTGCTCCCCGCCGCCGGC	X = 5-hydroxymethyl-2'-deoxycytidine
**o4499**	[6-FAM]GCCGGCGGCGGGGAGCAGCATGGAGCCTTXGGCTGACTGGCTGGCC	X = 5-methyl-2'-deoxycytidine

Table S5. ODNs used in the qPCR assay.

An asterisk in the oligonucleotide sequence indicates a 3'-5' phosphorothioate linkeage.

**o4627**	CTCTTCTGCCTGCTGACCTTTGtcagcCTTTCATTGATTGCGgattccaGAATTCACTAGTAAAGGAGAAGttgg ctacagcaa
**o4628**	CTCTTCTGCCTGCTGACCTTTGtcagcCTTTCATTGATTGCGcacgataGAATTCACTAGTAAAGGAGAAGgaac cgctcattg
**o4629**	CTCTTCTGCCTGCTGACCTTTGtcagcCTTTCATTGATTGCGtcacagaGAATTCACTAGTAAAGGAGAAGtgta gccctctgt
**o4368**	CTCTTCTGCCTGCTGACCTTTG
**o4371**	CTTTCATTGATTGCGgattccaGAATTCACTAGTAAAGGAGAAGttggctacagcaa
**o4372**	A*C*C*A*C*CCTGTTGCTGTAGCCAA
**o4373**	CTTTCATTGATTGCGcacgataGAATTCACTAGTAAAGGAGAAGgaaccgctcattg
**o4374**	C*A*C*C*A*TTGGCAATGAGCGGTTC
**o4392**	CTTTCATTGATTGCGtcacagaGAATTCACTAGTAAAGGAGAAGtgtagccctctgt
**o4393**	C*T*T*G*A*GCACACAGAGGGCTACA
**o4675**	[6-FAM]TCTTCXGTTTCCTCAGCXGAAGGCtcgagTCTTCXGTTTCCaagctTCAGCXGAAGGCTCTTCTGCCT GCTGACCTTTG X,X,X,X = 5-hydroxymethyl-2'-deoxycytidine
**o4677**	[6-FAM]TCTTCXGTTTCCTCAGCXGAAGGCtcgagTCTTCXGTTTCCaagctTCAGCXGAAGGCTCTTCTGCCT GCTGACCTTTG X,X,X,X = 5-methyl-2'-deoxycytidine
**o4727**	[Phos]CAAAGGTCAGCAGGCAGAAGAGCCTTXGGCTGAagcttGGAAAXGGAAGActcgaGCCTTXGGCTGAGG AAAXGGAAGA X,X,X,X = 5-methyl-2'-deoxycytidine
**o4728**	[6-FAM]TCTTCCGTTTCCTCAGCCGAAGGCtcgagTCTTCCGTTTCCaagctTCAGCCGAAGGCTCTTCTGCCT GCTGACCTTTG
**o4729**	[Phos]CAAAGGTCAGCAGGCAGAAGAGCCTTcGGCTGAagcttGGAAAcGGAAGActcgaGCCTTcGGCTGAGG AAAcGGAAGA
**o4123**	[Phos]TCAGCCTTTCATTGATTGCG
**o4124**	CTTCTCCTTTACTAGTGAATTC

Table S6. ODN pairs for FACS and EMSA. See Table S3 and Table S4 for ODN sequences.

Probe	FACS		EMSA	
binding trap	o2968	o2969	o2968	o2969
C/C	o3244	o3245	o2906	o2904
mC/C	o3081	o3245	o2967	o2904
hmC/C	o3211	o3245	o3115	o2904

fC/C	o3212	o3245	o3116	o2904
caC/C	o3213	o3245	o3117	o2904
mC/mC	o3081	o3214	o2967	o2909
hmC/mC	o3211	o3214	o3115	o2909
fC/mC	o3212	o3214	o3116	o2909
caC/mC	o3213	o3214	o3117	o2909
hmC/hmC	o3211	o3215	o3115	o3112
hmC/fC	o3212	o3215	o3116	o3112
hmC/caC	o3213	o3215	o3117	o3112
fC/fC	o3212	o3216	o3116	o3113
caC/fC	o3213	o3216	o3117	o3113
caC/caC	o3213	o3217	o3117	o3113

Table S7. Fit statistics for oligo-dA/dT context. Summary statistic of fits shown in Figure S5b–d.

Protein	Probe	Term	Estimate*	SEM	Statistic	*p* value
pBeB1859 (T/A/Y/N)	mC/mC	K_d1	79.61	15.02	5.30	5.17E-06
		B_max	0.67	0.03	20.94	1.82E-22
		bkg	0.08	0.01	5.93	7.02E-07
pBeB1859 (T/A/Y/N)	hmC/hmC	K_d1	384.46	99.59	3.86	3.85E-03
		B_max	0.55	0.06	9.83	4.11E-06
		bkg	0.09	0.01	9.82	4.16E-06
pBeB1859 (T/A/Y/N)	hmC/C	K_d1	1690	418	4.04	2.95E-03
		B_max	0.63	0.10	6.04	1.92E-04
		bkg	0.02	0.00	6.44	1.19E-04
pBeB1859 (T/A/Y/N)	hmC/mC	K_d1	8.49	1.68	5.05	5.64E-06
		B_max	0.67	0.03	23.28	1.69E-29
		bkg	0.07	0.03	2.68	9.76E-03
pBeB1859 (T/A/Y/N)	C/mC	K_d1	865	176	4.92	8.29E-04
		B_max	1.22	0.13	9.32	6.42E-06
		bkg	0.04	0.01	4.93	8.15E-04
pBeB2526 (T/C/Y/N)	mC/mC	K_d1	34.03	5.02	6.77	1.01E-07
		B_max	0.55	0.02	24.69	7.54E-23
		bkg	0.05	0.01	5.82	1.64E-06
pBeB2526 (T/C/Y/N)	hmC/hmC	K_d1	185	19.2	9.64	4.86E-06
		B_max	0.88	0.03	31.9	1.42E-10
		bkg	0.04	0.01	5.47	3.97E-04
pBeB2526 (T/C/Y/N)	hmC/C	K_d1	1642.65	399.54	4.11	2.63E-03
		B_max	1.10	0.18	6.21	1.57E-04
		bkg	0.02	0.00	5.15	6.02E-04
pBeB2526 (T/C/Y/N)	hmC/mC	K_d1	13.94	2.55	5.46	4.69E-06
		B_max	0.68	0.03	23.61	3.03E-22
		bkg	0.06	0.02	3.71	7.70E-04
pBeB2526 (T/C/Y/N)	C/mC	K_d1	1000	171	5.83	2.50E-04
		B_max	0.90	0.08	10.9	1.72E-06
		bkg	0.06	0.00	14.8	1.26E-07
pBeB1388 (wildtype)	mC/mC	K_d1	3.90	0.636	6.13	2.79E-04
		B_max	0.91	0.04	14.1	6.18E-07
		bkg	0.02	0.04	0.40	7.01E-01
pBeB1388 (wildtype)	hmC/hmC	K_d1	198	39.1	5.05	6.92E-04
		B_max	0.98	0.06	16.9	4.04E-08
		bkg	0.08	0.02	4.77	1.01E-03

pBeB1388 (wildtype)	hmC/C	K_d1	211	19.5	10.8	1.81E-06
		B_max	0.95	0.03	35.0	6.28E-11
		bkg	0.06	0.01	8.74	1.09E-05

(continued)

Table S7. (continued)

pBeB1388 (wildtype)	hmC/mC	K_d1	47.4	12.4	3.13	1.40E-02
		B_max	0.90	0.05	13.2	1.02E-06
		bkg	0.04	0.04	0.82	4.33E-01
pBeB1388 (wildtype)	C/mC	K_d1	43.8	3.64	12.0	7.54E-07
		B_max	0.93	0.16	58.6	6.15E-13
		bkg	0.03	0.01	2.50	3.37E-02

* K_d1 estimates in nM.

Table S8. Fit statistics for natural contexts. Summary statistic of fits shown in Figure S6.

Protein	Probe	Term	Estimate	SEM	Statistic	*p* value
pBeB1388 (wildtype)	Hey2	log10(K_d1x)	0.9395	0.2499	3.76	6.05E-04
		log10(K_d1y)	0.5952	0.2740	2.17	3.65E-02
		log10(K_d2)	2.6650	0.5159	5.17	9.04E-06
		B_max_x	1.0114	0.1557	6.49	1.53E-07
		bkg_x	0.2347	0.0727	3.23	2.67E-03
		B_max_y	0.7550	0.1236	6.11	4.99E-07
		bkg_y	0.0617	0.0830	0.74	4.62E-01
pBeB1859 (T/A/Y/N)	Hey2	log10(K_d1x)	0.3619	0.1192	3.04	4.26E-03
		log10(K_d1y)	1.3524	0.1508	8.97	5.06E-11
		log10(K_d2)	2.4846	0.2310	10.76	3.13E-13
		B_max_x	0.8268	0.0578	14.32	4.10E-17
		bkg_x	0.1072	0.0318	3.37	1.69E-03
		B_max_y	0.5118	0.0399	12.82	1.47E-15
		bkg_y	0.1141	0.0261	4.37	8.95E-05
pBeB1388	CDKN2A	log10(K_d1x)	1.2186	0.1184	10.29	6.33E-13
		log10(K_d1y)	1.8679	0.0790	23.65	1.64E-25
		log10(K_d2)	2.6988	0.3116	8.66	8.35E-11
		B_max_x	0.9547	0.1091	8.75	6.40E-11
		bkg_x	0.0752	0.0306	2.46	1.83E-02
		B_max_y	1.0647	0.1354	7.86	1.02E-09
		bkg_y	0.0700	0.0247	2.83	7.19E-03
pBeB1859	CDKN2A	log10(K_d1x)	1.6624	0.0806	20.61	2.98E-23
		log10(K_d1y)	1.1662	0.0819	14.24	1.80E-17
		log10(K_d2)	2.4576	0.1746	14.07	2.73E-17
		B_max_x	1.2733	0.0791	16.10	2.51E-19
		bkg_x	0.1538	0.0310	4.96	1.28E-05
		B_max_y	1.2693	0.0814	15.59	7.89E-19
		bkg_y	0.0858	0.0369	2.33	2.50E-02

* K_d1_x, K_d1_y, K_d2 estimates in nM. K_d1_x refers to the hmC/mC-modified duplex, K_d1_y to the mC/mC-modified duplex, K_d2 to other sequence features and is shared between both experimental conditions.

Methods

Molecular cloning for MBD cell-surface display and directed evolution. The entry vector for bacterial AIDA-I surface display, pBeB1383, was derived from the pET-21d(+) (Merck KGaA, Darmstadt, Germany) vector pBeB1380 (Ref.[2]) by replacing the N-terminal SpA(Z)-tag with the in silico-designed display cassette (o2912, FragmentGene, Genewiz, Leipzig, Germany; amplified with o3004 and o3005) using T4 ligation after *Nde*I and *Xho*I restriction of the plasmid backbone and *Nde*I and *Sal*I restriction of the display cassette (all enzymes purchased from New England Biolabs GmbH, Frankfurt am Main, Germany, "NEB" hereafter). This vector features a sequence encoding an N-terminal signal peptide (from *Vibrio cholerae* enterotoxin binding subunit CtxB) followed by an in-frame *Spe*I/*Xho*I cloning site, and sequences encoding the human c-Myc epitope tag, the *Escherichia coli* AIDA-I adhesin autotransporter linker, and the β-barrel domain (Figure S15, Table S2). Wildtype MBDs were subcloned from the expression vectors (see Ref.[2]) using *Spe*I/*Xho*I restriction-ligation cloning. Namely, "MeCP2" refers to hMeCP2[90–181] (CCDS14741.1), "MBD2" to hMBD2[146–225] (CCDS11953.1) and "MBD3" to hMBD3[2–81] (CCDS12072.1).

The NNK codon-degenerated MeCP2[K109X/V122X/Y123X/S134X] library was created by Gibson assembly of a double-stranded 118-mer oligodeoxynucleotide. To this end, the corresponding single-stranded 118-mer (o2901) was annealed at 2.0 µM to 2.5 µM o3111 (Figure S16) and incubated with 0.05 U/µL Klenow Fragment (3'-to-5' exo^{-}, NEB) in 50 mM Tris-HCl, 10 mM MgCl2, 1 mM ATP, 10 mM dithiothreitol, pH 7.5 (T4 DNA ligase buffer, NEB), supplemented with 0.1 mM of each dNTP (NEB) for 60 min at 37 °C. The reaction was quenched with 10 mM EDTA, concentrated by ice-cold ethanol precipitation and purified by silica column-chromatography (Macherey-Nagel, Düren, Germany). The AIDA-I surface display vector of wildtype MeCP2 (pBeB1570, derived from pBeB1380[2] as described above) was linearized in 35 cycles of a polymerase chain reaction (PCR) using 0.04 ng/µL plasmid, 2% (v/v) dimethyl sulfoxide, and 300 nM of each primer o2899 and o2900 in a KOD Hot Start DNA Polymerase PCR (Toyobo, Osaka, Japan) according to the manufacturer's instructions (66 °C annealing temperature) and purified without further ado like the 118-mer. 440 ng of the linearized backbone were assembled with 72 ng of the insert in a 20 µL NEBuilder HiFi DNA Assembly reaction

(NEB) at 45 °C for 40 min. The remainder PCR template was removed by *in situ*-digestion with 1 U/µL *Dpn*I at 37 °C for 60 min. After recovery by ethanol precipitation, the assembled library was transformed into electrocompetent DH10B (TOP10™☐, Thermo Fisher Scientific) at a target voltage of 1.8 kV over 4 ms. Transformation of 0.15 vol of the reaction per 100 µL competent cells (25 OD_{600}) typically yielded 400,000–800,000 colonies. This procedure was repeated until 8–9 million colonies had been recovered. The plasmid pool was purified from the spread-plated colonies using a commercial plasmid DNA purification protocol (Macherey-Nagel) and the degeneracy at the target sites confirmed by Sanger sequencing. The library was then transformed with 5- to 10-fold oversampling into electrocompetent Tuner™☐ (DE3) (Novagen, Merck Millipore). These strains were aliquoted and cryo-preserved at an OD600 of 3.0 in 15% glycerol at –80 °C until screening.

For recombinant expression, the surface-displayed MBD mutants could be sub-cloned by exchanging the MBD in an appropriate expression vector using *Spe*I/*Xho*I restriction-ligation cloning or by Gibson assembly using PCR amplification and *Xho*I-linearized entry vectors. Since the plasmid isolation from *E. coli* B strain Tuner™☐ (DE3) typically contained a substantial amount of genomic DNA impurities, we found the second approach superior in this case. We used pBeB1379 as entry vector for recombinant protein expression, which differs from pBeB1380 in the subject of the N-terminal solubility tag for the MBD, in this case, a FLAG epitope tag followed by maltose-binding protein (MBP). The fusion protein has a non-cleavable C-terminal 6xHis tag.

Molecular cloning of additional MBD variants. The non-binding MeCP2[R111A,R133A] mutants pJeJ2091 and pJeJ2370 were created by QuikChange™☐ site-directed mutagenesis (Agilent Technologies, Santa Clara, CA, U.S.) with o3996/o3997 and subsequently o3994/o3995. The A117C variants of pBeB1388 (MeCP2) and pBeB1859 (MeCP2[T/A/Y/N]) for EPR spin labeling were created using o4254/o4255 or o4280/o4281 in a QuikChange™☐ procedure respectively, yielding pBeB2573 and pBeB2577. The MeCP2 mutants T/C/Y/N and T/T/Q/K were retrieved by Gibson assembly as described above using o4053 (pBeB2526, pKaB2620) and o4054 (pBeB2527, pBeB2644).

Molecular cloning of MBD-GST fusion proteins. The GST tag from pGEX-6P1 (Merck) was amplified with o4763 and o4764 and introduced into the *Spe*I digested vectors pBeB1388 (MeCP2 wildtype), pBeB1859 (MeCP2[T/A/Y/N]) or pJeJ2091 (MeCP2[R111A,R133A]) by Gibson assembly as described above to yield pBeB2774, pBeB2775 and pBeB2776 respectively.

Strain validation. All strains were verified by Sanger DNA sequencing (Microsynth SeqLab, Göttingen, Germany).

Bacterial cell surface display of MBD proteins. *E. coli* B strain Tuner™☐ (DE3) (Novagen, Merck Millipore) harboring the desired surface-display vectors were inoculated from an over-night culture of a freshly transformed plate (monoclonal strains) or from 15% glycerol stocks (libraries, aliquoted at OD600 = 3.0) at a final OD600 of 0.05 in 1.5 mL (monoclonal strains) or 10 mL (libraries) LB with antibiotic, incubated at 220 rpm at 37 °C for 2.0–2.5 h and induced when their OD600 reached 0.4 to 0.6 with a final concentration of 50 µM IPTG. Higher IPTG concentrations lead to somewhat higher display levels, but also decreased survival after sorting. The cultures were then kept at 30 °C, 150 rpm for at least 1 h to maximum 2 h. The OD600 to harvest is dependent of the number of probes to measure per sample and the total number of events desired to screen. A 20 µL sample of the suspension prepared according to the following procedure typically yielded about 600,000 to 800,000 events: An OD600 of 0.8 was harvested (monoclonal strains), pelletized at 8,000 x g for 2 min and washed twice with PBS. The pellet was resuspended in 400 µL PBS or PBS containing 0.1% (m/v) ultra-pure bovine serum albumin (BSA; Cell Signaling Technology; a high purity was found to be crucial to minimize ghost detection while effectively preventing cells from sticking to each other). The suspension was then supplemented with 0.25 mM TCEP and incubated for 30–60 min on ice. In the meantime, the respective staining mixes were prepared (see below). 20 µL suspension (1.0 vol) was finally combined with 0.5 vol of the staining mix and incubated for 20–60 min at 22 °C, 700 rpm. Then, the bacteria were pelletized as described above and washed with 2.0 vol cold PBS (minimum 80 µL) to be finally re-suspended in 6.5 vol cold PBS (minimum 65 µL) and kept on ice until analysis. The

bacteria were analyzed on a SH800S Cell Sorter (Sony Biotechnology) using a 70 µm or 100 µm microfluidic sorting chip (Sony Biotechnology). The instrument was equipped with a 488 nm, 405 nm (not used), 638 nm and a 561 nm laser and the “Filter Pattern 2” was used to disentangle the collinear beams. AF488 fluorescence was detected in detector FL2 at 65% PMT, phycoerythrin (PE) in FL3 at 45% PMT and allophycocyanin (APC) in FL4 at 45% PMT. Regular cells from a control population were gated at a forward scatter intensity of 16 a.u. and 40% PMT of the backward scatter. Before loading, the samples were briefly resuspended by pipetting up and down and analyzed at c. 10,000 events per second or sorted at c. 100 events per second. Sorted cells were recovered in 1.5 mL reaction tubes pre-filled with super optimal broth (SOC) that contained the antibiotic. The sorted cells were kept on ice until the experiment was completed and then preferably spread-plated onto solid LB agar plates. However, outgrowth in liquid LB medium at 37 °C, 150 rpm over-night with immediate proceeding the following morning did not have noticeable downsides. Single cells were sorted directly onto one-well plates (Greiner Bio-One) containing solid LB agar with antibiotics and inoculated into liquid LB medium with antibiotic the next day. Glycerol stocks were prepared after the first outgrowth, preferably when the cultures had reached an OD600 of 4.5 by combining 700 µL culture with 300 µL 50% sterile-filtered glycerol. Overgrown cultures and clones kept at 4 °C over a prolonged time (longer than 1–2 weeks) can yield unsatisfactory results.

Preparation of FACS probes and staining mixes. dsDNA duplexes for the FACS binding assays (Table S6) were created by hybridization of two ssDNA probes that each are 5'-biotinylated. It was critical that the concentration of the probes was verified by spectrophotometry and adjusted at the indicated stages to allow for comparative assessment of binding selectivity. First, the ssDNA stocks were brought to a concentration of 50 µM in water. For oligo-dT probes we used an extinction coefficient at 260 nm of 197 mM^{-1} cm^{-1}, and for oligo-dA probes 286 mM^{-1} cm^{-1}. Then, 1 nmol of each ssDNA strand (20 µL) were combined and annealed in a total of 100 µL 2x EMSA buffer (10x buffer is 200 mM HEPES, 300 mM KCl, 10 mM EDTA, 10 mM $(NH_4)_2SO_4$, pH = 7.3), submerged in boiling water and brought slowly to room temperature. The concentration of this then 10 µM dsDNA stock was

measured and adjusted to 5 µM (A260 = 1.5) with water (ideally, 100 µL), aliquoted and stored at –20 °C. The FACS staining mix was composed of two components, A and B: Sufficient for 90 staining reactions (with 1.0 vol = 20 µL), component A was obtained by combining 270 µL 10x EMSA buffer and 54 µL 50 µM oligo(dA)·oligo(dT) competitor. Then, each 5 µM dsDNA probe was diluted in component A to 400 nM, e.g., for 6 staining reactions, 2.4 µL 5 µM dsDNA probe and 6.0 µL water were admixed to 21.6 µL component A. This mix should be kept at 4°C at all times. Component B contained the streptavidin (SAv)-fluorophore conjugate (SAv-PE, BioLegend; SAv-AF488, Thermo Fisher Scientific) and was prepared so that the SAv had a final concentration of 1.16 µM in 0.6 g/L BSA, 6 mM TCEP in ultrapure water. Component B was stable for up to two months when stored at 4 °C and shielded from light. After combining A, B and the cell suspension, the final staining reaction contained 0.08 OD600 cells, 66 nM dsDNA probe, 192 nM SAv-fluorophore conjugate(s) and, if present, 4 µM biotin as quencher in 1x EMSA buffer.

One-color DNA-binding FACS assay. To assess relative binding affinities, each 5 µM probe was diluted in component A (400 nM final dsDNA probe) and then an equal volume of component B was added by pipetting A into B. The pre-conjugation was allowed to proceed for 60 min on ice. Then, 20 µL (1.0 vol) cell suspension were combined with 10 µL (0.5 vol) of this mixture. The SAv-PE conjugated probes allowed a higher dynamic range to be assessed with this assay.

Two-color DNA-binding assay for screening on FACS. According to the screening goal, the staining mixes were prepared as described for the one-color DNA-binding FACS assay using different SAv-conjugates. It was convenient to prepare first a drop-out mix with equal volumes of the component A parts (with 400 nM dsDNA probe) that should be competed against, e.g., by combining the 14 "off-target" probes in component A, and then labeling 0.25 vol of this mix with 0.25 vol of component B (SAv-AF488). The desired "on-target" probe was then labeled as 0.05 vol to 0.05 vol of component B (SAv-PE). After both A–B-mixes were pre-conjugated, 0.05 vol 120 µM biotin was added to one of the A–B-mixes, that tube flicked, and incubated for another 5 min on ice. Then, both A–B-mixes were

combined (1/15 PE and 14/15 AF488), mixed by pipetting and 0.5 vol applied immediately to 1.0 vol cell suspension.

Determination of surface-display level by antibody staining. To determine the surface-display level of full-length payloads, the entry vector pBeB1383 had an in-frame c-myc epitope C-terminal of the *Spe*I/*Xho*I cloning site. The APC-coupled anti-myc antibody (Abcam) was diluted 1:5 in PBS and then 1.0 vol cell suspension combined with 0.5 vol of the dilute antibody. The labelling should be allowed to proceed for at least 60 min at room temperature, 700 rpm. We found that co-staining with the anti-myc antibody hampered detection of the DNA bound on the surface-displayed MBDs. As co-detection did only little help improving the false-positive rates during screening (high dispersion between DNA and antibody binding; further, no DNA binding was well identified in the two-color assay), we used the anti-myc staining only to discriminate unsuccessful binding events from unsuccessful displaying events in monoclonal strains.

Determination of false-positive rate of FACS screen. To determine the false-positive rate of the two-color DNA-binding assay for screening on FACS due to fluorophore migration, two-color assays were performed as described above using an equimolar mixture of a duplex containing a C/C CpG (o3244 + o3245) or a mC/mC CpG (o3801 + o3214) labelled in either SAv-fluorophore conjugate (PE or AF488, see above). Bacteria displaying MBD2 (pBeB1567) were used as positive control, the empty entry vector (pBeB1383) served as negative control. A total of 15,000 regular cells was sampled from the recorded data per experimental condition and the dependence of the fraction of double-positive events on the threshold levels determined in R.

Next-generation sequencing (NGS) after FACS screening. Glycerol stocks of the selected bacterial sub-libraries were inoculated in liquid LB medium with antibiotic and grown at 30 °C, 150 rpm to an OD600 of 4.0. The plasmids were then extracted using a commercial plasmid purification kit (Macherey-Nagel). For the initial degenerated MeCP2 library, the plasmid stock prior to transformation

in Tuner™☐ (DE3) was directly used as template. A 25 µL Phusion PCR (NEB) was used to amplify the degenerated region using o3861 and o3862 as primers which contained both degenerated hexamers (NNNNNN) as unique molecular identifiers (UMIs) and 50 ng plasmids in 25 cycles with an annealing temperature of 60 °C and 20 sec elongation. 2 µL of this reaction was then transferred to a fresh 25 µL Phusion PCR for barcoding the samples on both ends of the reads. Two independent dilutions of the initial MeCP2 library were subjected to this procedure. The samples were pooled according to clone number and sent for Illumina NGS to an external service provider (Genewiz Europe, Leipzig, Germany). The paired-end reads were merged using PANDAseq[5] (v2.11) and aligned with bbmap[6] (v36.86) in semiperfect mode to the reference which contained "NNN" at the positions of the degenerated codons, the position of the UMIs and the position of the barcodes of the second PCR amplification. The bam file was filtered, imported, and the sequences at the degenerated sites extracted using packages from the R Bioconductor suite[7,8]. Only reads were kept that had a mapping quality of 13 or higher and which showed the expected barcode pairs on both ends of their ends (ca. 70% of all reads). Distinct sequences were established based on UMIs and the codons present at the degenerated sites.

Sample	Physical clones	Glycerol stock ID	NGS barcode	Reads kept	Distinct sequences
library replicate 1	>1e6	–	BC08*	35,039	34,727
library replicate 2	>1e6	–	BC08*	37,323	37,008
strategy A sorting 2	250	F087	BC06	6,846	6,261
strategy B sorting 2	380	F107	BC04	793	725

* Retrieved in two different sequencing reactions of 50,000 reads each.

To calculate the abundance of a genotype or the respective phenotype, the fraction of UMI counts within the number of distinct sequences was used. For genotypes or phenotypes not present in the sequencing of the initial library (due to under sampling of more than 1 million clones with 2 x 50,000 reads), the original abundance was assumed to be 0.9 / (# distinct seq.), which is likely an

overestimate in most cases (Figure S4b). The amino acid enrichment per position was determined from the total of distinct codon–UMI combinations.

Recombinant protein expression. The proteins were recombinantly expressed in BL21-Gold(DE3) (Agilent Technologies) and 300 mL LB medium supplemented with 1 mM $MgCl_2$ and 1 mM $ZnSO_4$ and a suitable antibiotic inoculated from a fresh overnight culture. The culture was grown at 37 °C, 220 rpm shaking to an OD600 of 0.5–0.6, chilled on ice, and induced by supplying isopropyl β-D-1-thiogalactopyranoside (IPTG) to a final concentration of 1 mM (T7 *lacO* promoter system). The expression was allowed to proceed overnight at 30 °C, 150 rpm. The cultures were harvested at 8,000 x *g* for 20 min at 4 °C, washed twice by resuspension in 50 mL cold 20 mM Tris-HCl (pH 8.0), the supernatant removed and the pellet frozen until proceeding with the extraction. The pellet was resuspended in 20 mL binding buffer (20 mM Tris-HCl, 250 mM NaCl, 10% glycerol, 10 mM dithiothreitol, 5 mM imidazole, 0.1% Triton X-100, pH 8.0) supplemented with 1 mM phenylmethylsulfonyl fluoride and incubated overnight with a final concentration of 0.1 mg/mL lysozyme (Merck) and 1 U/mL DNase I (NEB). After two repeated sonication runs on ice (3 min per run of alternating a 4 sec ultrasonic wave pulse at 20% amplitude and 30 sec rest; Branson Digital Sonifier 450 Cell Disruptor), the cellular debris was removed by centrifugation at 14,000 x g for 20 min, 4 °C. The cleared supernatant was retained and purified over a Ni-NTA column (GE Healthcare) on an Äkta Purifier 10 FPLC system (GE Healthcare) using a gradient of imidazole (10 mM to 500 mM) in binding buffer. The fractions that contained the pure protein were combined and dialyzed three times against dialysis buffer (20 mM HEPES, 100 mM NaCl, 10% glycerol, adjusted to pH = 7.3, and 0.1% Triton X-100) using Slide-A-Lyzer dialysis cassettes (3.5 kDa MWCO, ThermoFisher Scientific, Waltham, MA, U.S.). The protein concentration was determined with a bicinchoninic acid assay (Pierce BCA Protein Assay Kit, ThermoFisher Scientific) in triplicates and the proteins stocked at 15 µM after snap freezing in liquid nitrogen, at –80 °C (stable for several months).

Large-scale protein expression for NMR spectroscopy. Starting from a 2 L expression culture (25 °C, 240 rpm, overnight) supplemented according to a protocol modified from Ref.[9] with ^{13}C glucose (Cambridge Isotope Laboratories, Andover, MA, U.S.) and ^{15}N-supplemented M9 medium, the bacterial pellet was resuspended in 30 mL extraction buffer and treated with lysozyme in presence of PMSF for 60 min on a wheelshaker at 4 °C. The suspension was extracted by pulse sonication or on a highshear microfluidizer homogenizer. Insoluble debris was removed at 30,000 × *g* for 45 min at 4 °C. The cleared supernatant was sterile-filtered (0.2 µm syringe filter) and loaded on a self-packed 10 mL column HisPurTM Ni-NTA Resin (Thermo Fisher) connected to an ÄKTA FPLC Fast Protein Liquid Chromatograph (GE Healthcare, Solingen, Germany). The mixture was separated at 1 mL/min flow rate of binding buffer containing 5 – 90 mM imidazole (0 – 100%) in 80 min. Fractions containing the MBP–MBD fusion protein were combined for dialysis. For spectroscopic NMR analyses, the solubility tag was cleaved by adding His-free TEV protease to the combined eluates of the column purification after a first dialysis (3.5 kDa MWCO) against binding buffer without imidazole (20 mL eluate against 2 L buffer). The dialysis buffer was exchanged once. Then, the His-free TEV and the solubility tag were removed over the same Ni-NTA column as before using a gradient of 0 – 30% over 150 min. The MBDs were eluted with 100% 90 mM imidazole in binding buffer and again the desired fractions combined. The combined fractions were concentrated over an Amicon® centrifugal filter device (3.5 kDa MWCO; Merck) to 1 – 2 mL for size-exclusion chromatography and loaded onto a HiPrep 26/60 Sephacryl® S-200HR dextran (Merck) column. The MBD domain was polished at a flow rate of 1 mL/min of the final buffer; the desired fractions combined and concentrated as before.

Electrophoretic-mobility shift assay (EMSA). EMSAs were carried out as described previously using the ODN pairs given in Table S6.[2] The MBP-tag was removed prior to the assay by treating a 15 µM MBD sample with 0.25 µM TEV protease (about 1 µg per mL aliquot) at 4 °C overnight.

Determination of binding affinity with single band shift. The gel shift data was quantified with ImageQuant TL v8.1 1D Gel Analysis (GE Healthcare) using rubber band background subtraction and

manual peak detection with approximately equal peak areas for each band across all lanes. The exported data was curated and analyzed with R v4.0.1 using the Levenberg-Marquardt nonlinear least-squares algorithm[10] to fit the following model:[11]

$$[RL] / [L]_0 = b + B \cdot ([R]_0 + [L]_0 + K_d - (([R]_0 + [L]_0 + K_d)^2 - 4[R]_0[L]_0)^{1/2}) / 2 / [L]_0$$

where $[L]_0$ is the total ligand concentration (2 nM) and $[R]_0$ the concentration of MBD in a given lane, b the background estimate and B a correction factor for variation in active probe constraint to $0.55 < B < 1.1$; $[RL] / [L]_0$ is the fraction of bound duplex per lane. Model estimates in Table S7. The fitting was implemented in the R package *summerrband* (DOI: 10.5281/zendo.5501758).

Determination of binding affinity with multiple band shifts. To evaluate gels that display multiple band shifts, the macroscopic apparent equilibrium constants for the association reaction K_1, K_2, … K_n were determined from the fitting of *n*-th degree binding isotherm:[12]

$$[RL] / [L]_0 = (K_1[R]_0 + 2K_1K_2[R]_0^2 + \ldots + nK_1 \cdots K_n[R]_0^n) / (1 + K_1[R]_0 + K_1K_2[R]_0^2 + \ldots + K_1 \cdots K_n[R]_0^n)$$

If the microscopic binding constant k_a for occupying the first (most affine) site *a* is sufficiently different from binding the second, third or further sites *b*, *c*, etc., then K_1 is a good estimator for k_a given there is no or negligible little cooperation between the binding sites (which is reasonable for MBD binding in this case). The fitting was implemented such that all $K_{n > 1}$ were shared for DNA probes with the same sequence (representing the same additional binding sites *b*, *c* etc.) and only the K_1 were specific for the differentially modified CpGs. Model estimates in Table S8. This (and some additional microscopic model fitting) were implemented in *summerrband* (DOI: 10.5281/zendo.5501758).

Site-directed spin labeling of proteins. The protein constructs containing engineered cysteines were thawed and incubated with a 3-fold molar excess of Tris-(2-carboxyethyl)-phosphin (TCEP, Sigma Aldrich) at room temperature for 30 min. Afterwards, TCEP was removed using Zeba™ Spin Desalting Resin (7 MWKO, Thermo Fisher). A 6-fold molar excess of (1-Oxyl-2,2,5,5-tetramethylpyrroline-3-methyl) methanethiosulfonate (MTSL, Enzo Life Sciences) in DMSO was added to the protein samples and incubation was performed over night at 4°C and 300 rpm (ThermoMixer C, Eppendorf).

The next day, excess label was removed via diafiltration (Amicon Ultra 0.5 mL Centrifugal Filters, Merck Millipore). Protein concentration after labeling was determined using biochemical methods (BCA Protein Assay Kit, Thermo Fisher). Labeling efficiencies were determined as ratio between number of spins (Xenon Nanon Software Package, EMXnano Spectrometer, Bruker) and protein concentration (quantitative labeling). Additionally, mass spectra were acquired to determine sample composition (ESI-MS, amazon speed ETD, Bruker). Samples were flash frozen in liquid nitrogen and stored at -80°C until further usage.

Site-directed spin labeling of DNA oligonucleotides. The phosphorothioate-containing single-stranded oligonucleotides o4329 and o4329 were spin labeled according to published procedure.[13] Briefly, 2.48 mg of 1-Oxyl-2,2,5,5-tetramethyl-3(methanesulfonyloxymethyl)pyrroline (compound 1, Toronto Research Chemicals) was dissolved in 500 µL dry acetone and 10 µL of 1 M NaI stock solution in dry acetone was added. The mixture was incubated for 60 min at 37°C and 300 rpm (ThermoMixer C, Eppendorf). White precipitate of $NaOSO_2CH_3$ formed while successful reaction was separated from 3-Iodomethyl-1-oxyl-2,2,5,5-tetramethylpyrroline (compound 2) via centrifugation at 18.000 x g for 10 min. The precipitate was washed once with dry acetone and centrifugation was repeated. The supernatants were combined and the solvent was removed using a constant flow of nitrogen. Afterwards, compound 2 was dissolved in 20 µL dry acetonitrile and mixed with 10 µL of 1 M MES buffer pH 5.8 (Roth), 45 µL of 400 µM oligo stock (o4329 or o4328) and 25 µL milliQ. The mixture was incubated at 21°C and 700 rpm (ThermoMixer C, Eppendorf) overnight in the dark. The reaction was stopped and the mixture was stored at -20°C until further purification. The labeled oligonucleotides were purified by anion-exchange HPLC using a Dionex PA-100 column and applying a step-gradient for elution (Buffer A: 1 mM NaClO4, 20 mM Tris–HCl (pH 6.8), 20% (v/v) acetonitrile, Buffer B: 400 mM NaClO4, 20 mM Tris–HCl (pH 6.8), 20% (v/v) acetonitrile). Sample fractions were chosen according to HPLC chromatogram and combined. The HPLC-fractions were desalted using G-25 Sephadex columns (GE Healthcare) with milliQ as running buffer. The eluate was freeze-dried and resuspended in milliQ. Concentrations of the labeled oligonucleotide samples were determined via absorbance at 260 nm (BioPhotometer D30, Eppendorf). The amount of spins was determined using the Xenon Nano software package (EMXnano Spectrometer, Bruker). Respective yields and labeling efficiencies were 5.60 nmol (42.1 µM spin concentration; >100% labeling efficiency) for o4329 and 9.74 nmol (56.4 µM spin concentration; 96% labeling efficiency).

To generate the double-stranded DNA duplexes, the desired amount of matching single-strand oligonucleotides (mC/mC-dA*: o4329/o2909, mC/mC-dT*: o2967/o4328, hmC/mC-dT*: o3115/o4328) were mixed in 30 mM HEPES, 100 mM KOAc and heated to 95°C for 5 min (ThermoMixer C, Eppendorf). The mixture was slowly cooled to room temperature and freeze-dried. The final double-

stranded DNA hybrids were dissolved in desired amount of H_2O or D_2O and stored at 4 °C until further usage.

Double electron-electron resonance (DEER) experiments. 10 μM double-stranded DNA and protein (ratio 1:1.2 or 1:2) were mixed and incubated for 20 min at 22 °C (ThermoMixer C, Eppendorf). Afterwards, 50% (v/v) d_8-glycerol (Sigma) was added to yield a final volume of 60 μL. The samples were transferred into 3 mm outer diameter quartz tubes (Fused quartz tubing, Technical Glass Products) and flash frozen in liquid nitrogen. All experiments were conducted at Q-band frequency (34 GHz) and 50 K with an Elexsys E580 spectrometer (Bruker) equipped with an arbitrary waveform generator (AWG) unit (Bruker) and a 150 W traveling-wave tube (TWT) amplifier (Applied Systems Engineering) in a commercial Q-band resonator (ER5106QT-2, Bruker). The temperature was controlled by a cryogen-free helium recirculation system with a ColdEdge cryocooler (CE-FLEX-4K-0110, Bruker), a F-70H helium compressor (SHI cryogenics), and a MercuryiTC temperature controller (Oxford Instruments). All experiments were performed with the standard four-pulse DEER sequence ($\pi/2_{obs} - \tau_1 - \pi_{obs} - (\tau_1 + t) - \pi_{pump} - (\tau_2 - t) - \pi_{obs} - \tau_2$ – echo)[14] with rectangular pump and observer pulses. The pump frequency was set to 34 GHz. A frequency offset of 70 MHz (33.93 GHz) was chosen for the observer pulse. The length of the pump pulse was set to 16 ns and 24 ns for the observer pulse was used. Nuclear modulation averaging was done by increasing τ_1 in 8 steps by 16 ns each. Artefacts caused by interfering echoes were removed with an 8-step on pump {(x) x [xp] x} phase cycling.[15] Procession and analysis of raw DEER data was performed with MatLab R2019b (The MatWorks, Inc.), the toolbox EasySpin 6.0.0[16] and DeerAnalysis 2019[17]. Distance distributions were extracted using an experimental background function followed by Tikhonov regularization using the L-curve criterion. Distributions were validated (prune level 1.15) by variation of the background start (typically in a range of 1600 ns around the chosen background start / 50 steps). Expected distance distributions were based on simulations (MMM version 2018.2).[18]

NMR sample preparation and measurement. For NMR studies, uniformly $^{13}C/^{15}N$-doubly-labeled protein was produced in minimal (M9) media supplemented with $^{15}NH_4Cl$ and ^{13}C-glucose as the sole source of nitrogen and carbon, respectively and purified (see "Large-scale protein expression"). Purified uniformly-$^{13}C/^{15}N$ MeCP2 and its triple-mutant were prepared in a mixed solvent of 90% H_2O and 10% ^2H_2O (50 mM sodium phosphate, 50 mM NaCl, pH = 6). All NMR experiments were carried out at 18 ºC with protein concentrations of 0.5 mM on a Bruker Avance 800 MHz NMR spectrometer equipped with a 5 mm cryogenically cooled triple-resonance probe and a pulse-field gradient. A suite of standard 3D double- and triple-resonance NMR experiments were performed for sequence-specific ^1H, ^{13}C and ^{15}N backbone resonance assignments as discussed earlier.[19] The ^1H

chemical shifts were referenced to the external standard 2, 2-dimethyl-2-silapentene-5-sulfonates (DSS), while ^{15}N and ^{13}C chemical shifts were calibrated indirectly. The near-complete ^{1}H, ^{13}C and ^{15}N resonance assignments of mutant MeCP2 protein has been deposited to the BMRB (http://www.bmrb.wisc.edu) under the accession number 51020. A similar sequence for the wildtype protein can be found under accession code 4280. The chemical-shift perturbations (CSPs) were measured as $[(\Delta H)^2+(\Delta N/10)^2]^{1/2}$, where ΔH and ΔN signify the changes in $^{1}H^{N}$ and ^{15}N chemical shifts, respectively. The factor 10 for ^{15}N chemical shift was taken as the normalization factor since the broad range of nitrogen chemical shifts is approximately ten times that of proton chemical shifts for the backbone amides in folded proteins.

Neighbor-corrected structural propensity and chemical-shift perturbation calculation. Secondary structural elements of both proteins were assessed via NMR chemical shifts of ^{15}N, $^{13}C^{\alpha}$, $^{13}C^{\beta}$, $^{1}H^{H}$, and ^{13}CO in the framework of neighbor-corrected structural-propensity prediction.[20]

Preparation of spike-in probes. Spike-in probes are ligation products of a DNA duplex carrying the modified CpGs ("carrier") and a DNA duplex with a general and a unique primer binding site for quantitation by qPCR ("adapter"). Adapters were created by primer extension of the primer pairs o4371/o4372, o4373/o4374, and o4392/o4393 (Table S5) annealed at 2.5 µM in 1x T4 DNA ligase buffer (NEB) and elongated (blunted) in presence of 0.1 mM dNTPs and 25 mU/µL (large) Klenow fragment of DNA polymerase I (NEB) at 37 °C for 20 min. The double-stranded products were 5'-phosphorylated repeating the procedure in situ with a 2-fold excess of o4123. The crude reaction provided the buffer conditions for ligation of the carriers. Carriers were prepared by annealing the unmodified or modified oligonucleotides (C/C: o4728/o4729, mC/mC: o4677/o4727, hmC/mC: o4675/o4727) at 5.0 µM in 30 mM HEPES, 100 mM KOAc, pH = 7.5 (extinction coefficient of the duplex was 1011 mM^{-1} cm^{-1}). Then, 15 pmol carrier were added to 50 µL of the crude adapters (enabling different "qPCR barcoding" combinations) and ligated with 200 U T4 DNA ligase (NEB) at 16 °C overnight. Excess adapters were removed by column purification using a commercial kit with an

appropriate dilution of the chaotropic agent (here: 16% NTI in water, Macherey-Nagel) and completion of the reaction confirmed by agarose gel electrophoresis. Since the remaining traces of adapters render quantitation by UV/Vis photospectroscopy unreliable, the amount of functional product must be quantitated using a qPCR dilution series with the universal reverse primer in reference to which the efficiency of amplification with the specific primers can be established. qPCR conditions and absolute concentration references were optimized using synthetic oligonucleotides that contained both primer binding sites (o4627 for o4372, o4628 for o4374, o4629 for o4393). The optimal qPCR conditions were 600 nM o4368 and 600 nM of the specific reverse primer (o4372, o4374, o4393) or the universal reverse primer o4124 using 5 µL 2 x primaQUANT SYBRGreen Master Mix with ROX (Steinbrenner Laborsysteme GmbH, Wiesenbach, Germany), 3 µL primer mix 2 µM primer mix (each), 2 µL template and qPCR amplification after an initial denaturation at 95 °C for 2 min by 45–50 cycles of 95 °C for 10 sec, 60 °C for 30 sec. The lowest concentration that could be reliably quantitated in presence of 200 pM off-target were >1.2 pM (o4372), >16 fM (o4374) and >0.16 fM (o4393).

Enrichment of spike-in probes from whole-genome amplificated DNA or HEK293T genomic DNA. Two sourced of DNA background were used: Either genomic DNA isolated from HEK293T cells maintained under standard culturing conditions isolated with a commercial kit (QIAmp DNA Mini kit; Qiagen, Hilden, Germany) or whole-genome amplified DNA from human genomic DNA (male Yoruban individual, Encode entry NA18507, Coriell Institute, Camden, NJ, U. S.) using illustra Ready-To-Go GenomiPhi V3 whole genome amplification kit (GE Healthcare) to deplete DNA modifications. Background DNA was sheared to an average length of 200 bp in a Bioruptor Pico (Diagenode). Per enrichment reaction, spike-in probes were pooled at 4.62 fmol (50,000 copies each) or 0.462 fmol (5,000 copies; each) in 200 ng sheared DNA. The manufacturer's protocol (MethylCap kit; Diagenode, Seraing, Belgium) was followed at 0.2 vol scale with minor adjustments: The total amount per reaction was 200 ng DNA containing the spike-ins in 23.8 µL "Buffer B" and 2 µL of 1:5 in dialysis buffer diluted ~100 µM MBD–GST stock, i.e., 2 µL of a ~20 µM MBD–GST stock (MethylCap kit protein, p2774, p2775, or p2776; the MBP tag of the latter three was removed by TEV cleavage in situ for 30 min at

room temperature, then placed on ice; the MethylCap kit protein was treated likewise). The capture reaction was allowed to take place over 2 hours (can be performed also overnight) in 200 µL tubes in a rotating wheel at 4 °C before 10 µL of the supplied GSH-coated magnetic beads were added. The MBD-DNA complexes were immobilized for 1 h. Then, each reaction was washed with 1 x 40 µL "Wash Buffer 1" and 2 x 40 µL "Wash Buffer 2" for 5 min shaking at 950 rpm at 16 °C before the DNA was recovered at once in 30 µL "High Elution Buffer" for 10 min. The supernatant (or 10% input diluted in elution buffer) was column-purified using a commercial kit (Macherey-Nagel, Düren, Germany) and recovered in 2 x 20 µL (50,000 copies samples) or 2 x 10 µL (5,000 copies samples) 5 mM Tris-HCl pH = 8.5. qPCR measurements were made in duplicates for each sample and each target (optimized conditions see "Preparation of spike-in probes"). Individual spike-in concentrations were determined relative to a dilution series of pure spike-in probes and the recovery determined relative to the input.

References

(1) Hashimoto, H.; Liu, Y.; Upadhyay, A. K.; Chang, Y.; Howerton, S. B.; Vertino, P. M.; Zhang, X.; Cheng, X. Recognition and Potential Mechanisms for Replication and Erasure of Cytosine Hydroxymethylation. *Nucleic Acids Res* **2012**, *40* (11), 4841–4849. https://doi.org/10.1093/nar/gks155.

(2) Buchmuller, B. C.; Kosel, B.; Summerer, D. Complete Profiling of Methyl-CpG-Binding Domains for Combinations of Cytosine Modifications at CpG Dinucleotides Reveals Differential Read-out in Normal and Rett-Associated States. *Sci Rep* **2020**, *10* (1), 4053. https://doi.org/10.1038/s41598-020-61030-1.

(3) Nakata, M.; Zanchetta, G.; Chapman, B. D.; Jones, C. D.; Cross, J. O.; Pindak, R.; Bellini, T.; Clark, N. A. End-to-End Stacking and Liquid Crystal Condensation of 6– to 20–Base Pair DNA Duplexes. *Science* **2007**, *318* (5854), 1276–1279. https://doi.org/10.1126/science.1143826.

(4) Krstić, I.; Hänsel, R.; Romainczyk, O.; Engels, J. W.; Dötsch, V.; Prisner, T. F. Long-Range Distance Measurements on Nucleic Acids in Cells by Pulsed EPR Spectroscopy. *Angewandte Chemie Int Ed* **2011**, *50* (22), 5070–5074. https://doi.org/10.1002/anie.201100886.

(5) Masella, A. P.; Bartram, A. K.; Truszkowski, J. M.; Brown, D. G.; Neufeld, J. D. PANDAseq: Paired-End Assembler for Illumina Sequences. *Bmc Bioinformatics* **2012**, *13* (1), 31. https://doi.org/10.1186/1471-2105-13-31.

(6) Bushnell, B. BBMap short read aligner. https://sourceforge.net/projects/bbmap/ (accessed 2019 - 08 -01).

(7) Lawrence, M.; Huber, W.; Pagès, H.; Aboyoun, P.; Carlson, M.; Gentleman, R.; Morgan, M. T.; Carey, V. J. Software for Computing and Annotating Genomic Ranges. *Plos Comput Biol* **2013**, *9* (8), e1003118. https://doi.org/10.1371/journal.pcbi.1003118.

(8) Pagès, H.; Aboyoun, P.; Gentleman, R.; DebRoy, S. *Biostrings: String Objects Representing Biological Sequences, and Matching Algorithms. R Package Version 2.40.2*; 2016.

(9) Marley, J.; Lu, M.; Bracken, C. A Method for Efficient Isotopic Labeling of Recombinant Proteins. *J Biomol NMR* **2001**, *20* (1), 71–75. https://doi.org/10.1023/a:1011254402785.

(10) Elzhov, T. V.; Mullen, K. M.; Spiess, A.-N.; Bolker, B. Minpack.Lm: R Interface to the Levenberg-Marquardt Nonlinear Least-Squares Algorithm Found in MINPACK, Plus Support for Bounds. 2016.

(11) Altschuler, S. E.; Lewis, K. A.; Wuttke, D. S. Practical Strategies for the Evaluation of High-Affinity Protein/Nucleic Acid Interactions. *J Nucleic Acids Investigation* **2012**, *4* (1), 3. https://doi.org/10.4081/jnai.2013.e3.

(12) Ben-Naim, A. The Binding Isotherm. In *Cooperativity and Regulation in Biochemical Processes*; Springer: Boston, MA, 2001; pp 25–49. https://doi.org/10.1007/978-1-4757-3302-0_2.

(13) Qin, P. Z.; Haworth, I. S.; Cai, Q.; Kusnetzow, A. K.; Grant, G. P. G.; Price, E. A.; Sowa, G. Z.; Popova, A.; Herreros, B.; He, H. Measuring Nanometer Distances in Nucleic Acids Using a Sequence-Independent Nitroxide Probe. *Nat Protoc* **2007**, *2* (10), 2354–2365. https://doi.org/10.1038/nprot.2007.308.

(14) Pannier, M.; Veit, S.; Godt, A.; Jeschke, G.; Spiess, H. W. Dead-Time Free Measurement of Dipole–Dipole Interactions between Electron Spins. *J Magn Reson* **2011**, *213* (2), 316–325. https://doi.org/10.1016/j.jmr.2011.08.035.

(15) Tait, C. E.; Stoll, S. Coherent Pump Pulses in Double Electron Electron Resonance Spectroscopy. *Phys Chem Chem Phys* **2016**, *18* (27), 18470–18485. https://doi.org/10.1039/c6cp03555h.

(16) Stoll, S.; Schweiger, A. EasySpin, a Comprehensive Software Package for Spectral Simulation and Analysis in EPR. *J Magn Reson* **2006**, *178* (1), 42–55. https://doi.org/10.1016/j.jmr.2005.08.013.

(17) Jeschke, G.; Chechik, V.; Ionita, P.; Godt, A.; Zimmermann, H.; Banham, J.; Timmel, C. R.; Hilger, D.; Jung, H. DeerAnalysis2006—a Comprehensive Software Package for Analyzing Pulsed ELDOR Data. *Appl Magn Reson* **2006**, *30* (3–4), 473–498. https://doi.org/10.1007/bf03166213.

(18) Jeschke, G. MMM: A Toolbox for Integrative Structure Modeling. *Protein Sci* **2018**, *27* (1), 76–85. https://doi.org/10.1002/pro.3269.

(19) Wakefield, R. I. D.; Smith, B. O.; Nan, X.; Free, A.; Soteriou, A.; Uhrin, D.; Bird, A. P.; Barlow, P. N. The Solution Structure of the Domain from MeCP2 That Binds to Methylated DNA. *J Mol Biol* **1999**, *291* (5), 1055–1065. https://doi.org/10.1006/jmbi.1999.3023.

(20) Tamiola, K.; Mulder, F. A. A. Using NMR Chemical Shifts to Calculate the Propensity for Structural Order and Disorder in Proteins. *Biochem Soc T* **2012**, *40* (5), 1014–1020. https://doi.org/10.1042/bst20120171.
